# A highly specific phage defense system is a conserved feature of the *Vibrio cholerae* mobilome

**DOI:** 10.1371/journal.pgen.1006838

**Published:** 2017-06-08

**Authors:** Brendan J. O’Hara, Zachary K. Barth, Amelia C. McKitterick, Kimberley D. Seed

**Affiliations:** 1 Department of Molecular, Cellular and Developmental Biology, University of Michigan, Ann Arbor, Michigan, United States of America; 2 Department of Plant and Microbial Biology, University of California, Berkeley, Berkeley, California, United States of America; North Carolina State University, UNITED STATES

## Abstract

*Vibrio cholerae*-specific bacteriophages are common features of the microbial community during cholera infection in humans. Phages impose strong selective pressure that favors the expansion of phage-resistant strains over their vulnerable counterparts. The mechanisms allowing virulent *V*. *cholerae* strains to defend against the ubiquitous threat of predatory phages have not been established. Here, we show that *V*. *cholerae* PLEs (phage-inducible chromosomal island-like elements) are widespread genomic islands dedicated to phage defense. Analysis of *V*. *cholerae* isolates spanning a 60-year collection period identified five unique PLEs. Remarkably, we found that all PLEs (regardless of geographic or temporal origin) respond to infection by a myovirus called ICP1, the most prominent *V*. *cholerae* phage found in cholera patient stool samples from Bangladesh. We found that PLE activity reduces phage genome replication and accelerates cell lysis following ICP1 infection, killing infected host cells and preventing the production of progeny phage. PLEs are mobilized by ICP1 infection and can spread to neighboring cells such that protection from phage predation can be horizontally acquired. Our results reveal that PLEs are a persistent feature of the *V*. *cholerae* mobilome that are adapted to providing protection from a single predatory phage and advance our understanding of how phages influence pathogen evolution.

## Author summary

*Vibrio cholerae* is the causative agent of the severe diarrheal disease cholera. *V*. *cholerae* is commonly recovered from patient samples with predatory bacteriophages (phages), which impose strong selective pressure favoring phage resistant strains over their vulnerable counterparts. Here, we investigated the activity of PLEs (phage-inducible chromosomal island-like elements), a novel group of mobile genetic elements that have contributed to phage resistance in *V*. *cholerae* over the last 60 years. Surprisingly, we found that PLEs are protective against a single, prevalent phage type. We found that PLE activity reduces phage genome replication and accelerates the kinetics of bacterial cell lysis. Our study shows that mobile genetic elements play a key role in phage resistance in successful epidemic *V*. *cholerae*.

## Introduction

A chief determinant of microbial survival is protection from predation. Phages are viral predators that act with exquisite specificity to kill their perpetually evolving bacterial targets. The overall success of epidemic *Vibrio cholerae*, the causative agent of the diarrheal disease cholera, is partly due to its ability to defend against predatory phages. Such phages are found in the aquatic environment [1] and are co-ingested with *V*. *cholerae*, permitting continued phage predation of *V*. *cholerae* within the human intestinal tract [2]. Recent molecular characterization of lytic phages associated with epidemic cholera has revealed that phage diversity is strikingly low over significant time periods, indicating that a surprisingly limited number of phage types place a significant predatory burden on *V*. *cholerae* in the context of human infection [2,3]. The most prominent phage found with *V*. *cholerae* in cholera patient stool in the endemic region of Bangladesh are the ICP1-related virulent (lytic) myoviruses [3]. ICP1 uses the lipopolysaccharide O1 antigen of *V*. *cholerae* to bind to cells and initiate infection [3]. The O1 antigen is required for *V*. *cholerae* to efficiently colonize the small intestine [4], which places mutational constraints on *V*. *cholerae* in the human host and ensures ICP1 has access to susceptible *V*. *cholerae* in order to propagate [5].

Bacteria have evolved diverse antiviral resistance strategies to defend against the ubiquitous threat of predatory phages [6]. As obligate bacterial parasites, phages counter-adapt to overcome these resistance barriers, resulting in a dynamic co-evolutionary arms race [7]. The pervasiveness of ICP1 in Bangladesh with continued cholera epidemics suggests that *V*. *cholerae* has strategies to limit ICP1 predation that do not compromise virulence, and that ICP1 can evolve to overcome such defenses. Comparisons between sequenced ICP1 isolates revealed that roughly half of all ICP1 isolates encode a functional CRISPR–Cas (clustered regularly interspaced short palindromic repeats–CRISPR-associated proteins) system [8]. CRISPR–Cas systems function as adaptive immune systems that utilize small effector RNAs in complex with Cas proteins to direct the sequence specific degradation of invading DNA [9,10]. Typically, bacteria employ CRISPR–Cas to target invading phage DNA, therefore the ICP1 phage-encoded CRISPR–Cas system is a unique example of the unexpected genetic novelty found in studying phage-host coevolution. The ICP1 phage-encoded CRISPR–Cas system is utilized to mediate the degradation of a phage-inhibitory chromosomal island encoded by *V*. *cholerae* referred to as a phage-inducible chromosomal island-like element (PLE) [8]. The nature of how the PLE protects *V*. *cholerae* from infection by CRISPR–Cas deficient phage has not been described.

PLEs have no sequence similarity to other known anti-phage systems; however, PLE 1’s designation was based on evidence that this island functionally resembles phage-inducible chromosomal islands (PICIs) of Gram-positive bacteria [8]. The staphylococcal pathogenicity islands (SaPIs) are well studied PICIs that take advantage of helper phages to enable their own replication and spread [11,12]. SaPIs are named for their role in pathogenesis, as they carry genes encoding for toxic shock syndrome toxin and other superantigens [13]. SaPIs exist quiescently in their host’s chromosome and are induced to excise and replicate upon initiation of their temperate helper phage’s lytic cycle. The SaPI life cycle results in the packaging of the SaPI genome into infectious phage-like transducing particles that permit horizontal spread of the SaPI. SaPIs use structural gene products encoded by the helper phage for their encapsidation [14,15]. SaPI mobilization, however, interferes with helper phage replication, a phenotype typified by their ability to inhibit helper phage plaque formation [16–18]. Like the SaPIs, *V*. *cholerae* PLE 1 inhibits plaque formation by ICP1 in the absence of ICP1 phage-encoded CRISPR targeting, and PLE 1 excises in response to ICP1 infection [8].

Here, we used bioinformatic approaches to identify PLEs in a geographically and temporally diverse collection of *V*. *cholerae* isolates. We discovered a total of five PLEs and found that a conserved feature of these islands is their ability to interfere with ICP1 phages. PLE activity abolishes ICP1 proliferation, and while we were unable to recover phage mutants that escape PLE-mediated interference in experimental evolution experiments, we found that ICP1 isolates recovered from patient samples display unique susceptibility patterns to different PLEs. We show that PLEs, like SaPIs, are mobilized in response to phage infection and can spread to neighboring cells such that protection from phage predation can be horizontally acquired. We demonstrate that phage genome replication is inhibited by PLE activity and cell lysis is accelerated following ICP1 infection when PLE is active, indicating a multi-faceted mode of phage interference. Together, our results reveal the significance of a specific predatory phage in the evolutionary history of epidemic *V*. *cholerae* and provide new insight into mechanisms underpinning phage-host coevolution.

## Results

### PLEs are a persistent feature of the *V*. *cholerae* mobilome

**Fig 1:**
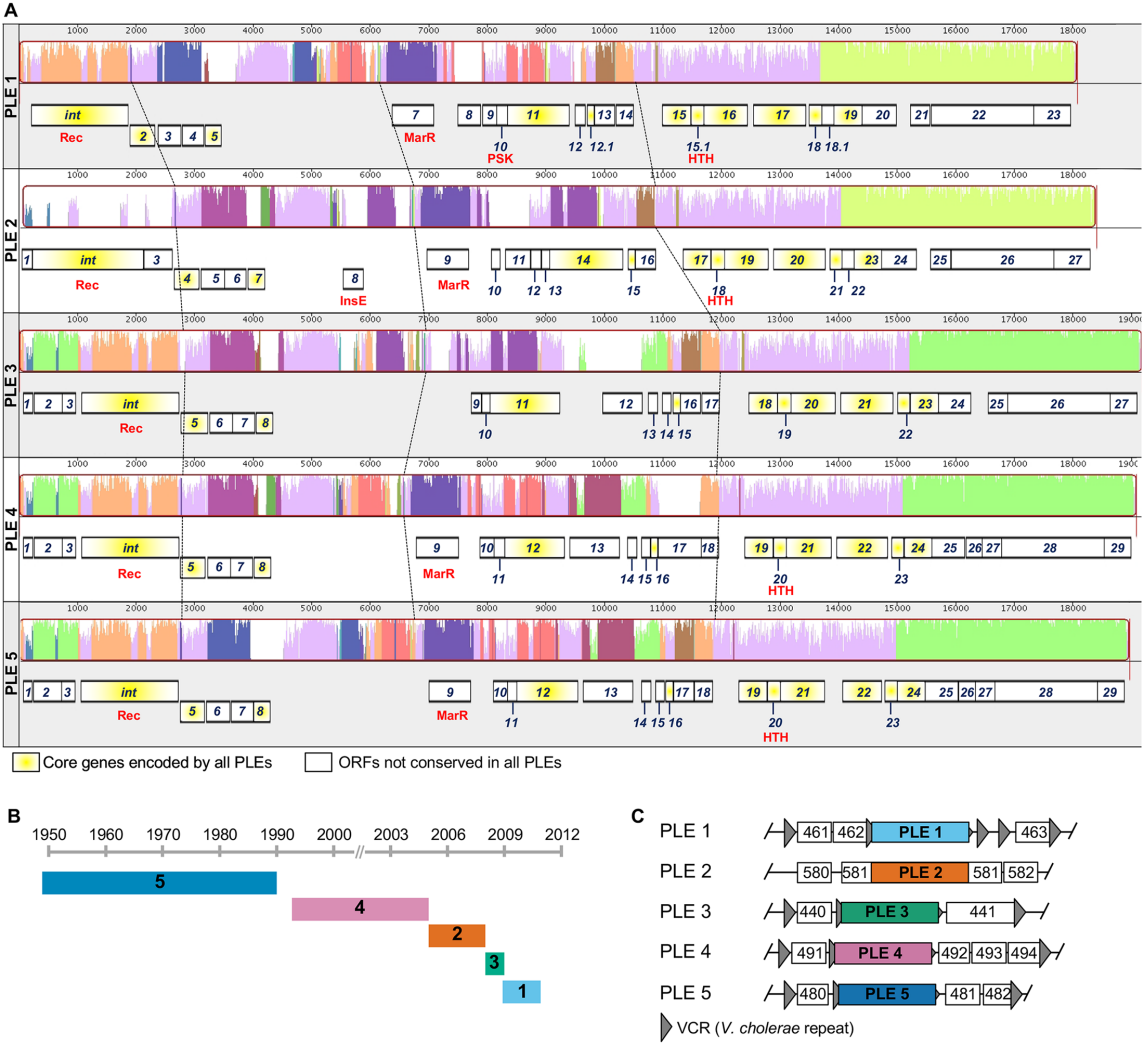
Conserved PLEs are a persistent feature of the *V*. *cholerae* mobilome. (A) Genomic organization and alignment of *V*. *cholerae* PLEs. Alignments were performed in MAUVE using the progressiveMAUVE algorithm [35]. Parts of the similarity plot that are colored lavender are conserved among all five genomes, with the height of the histogram representing nucleotide sequence identity. Regions conserved only among subsets of the PLEs are color coded differently. White regions correspond to unaligned sequences that contain sequence elements specific to each PLE. The dashed lines indicate regions in each PLE with shared sequence identity and serve as orientation points. Annotated genes are shown to scale as black outlined boxes, with genes transcribed from the reverse strand shifted downward. The integrase (*int*) and genes encoding hypothetical proteins (with numerical ORF designations) are indicated, and those with conserved domains are identified in red as described in the text. Core proteins [36] encoded by all PLEs are indicated in yellow. (B) History of PLE prevalence in >200 *V*. *cholerae* strains isolated between 1949–2011 [19,20]. The date range indicated represents the earliest and latest isolation of a given PLE^+^ isolate, and the number corresponds to the numerical PLE designation in panel A. (C) PLEs are found integrated into the *V*. *cholerae* small chromosome. ORFs are indicated by white boxes with 3 digit numbers corresponding to the VCA0XXX designation as observed in the N16961 reference genome. The flanking genes indicate the position of each PLE in clinical isolates. The positions of VCRs in the immediate vicinity of each PLE (if applicable) are shown. Diagram is not to scale.

By analyzing the genomes of >200 *V*. *cholerae* isolates with known geographic and temporal origins [19,20] we identified five unique PLEs, each predicted to encode up to 29 open reading frames (ORFs) (Fig 1A). A nucleotide alignment of *V*. *cholerae* PLEs shows that PLEs are void of genomic rearrangements. At the protein level, PLEs encode a conserved set of eleven predicted proteins (protein translations for all predicted PLE-encoded ORFs are found in S1 Dataset). In silico analyses (using CDD [21], Pfam [22] and BLASTp) of PLE proteins revealed that only five proteins have conserved domains shared with known proteins (e-value < 1 × 10^−2^). All PLEs encode an integrase with a serine recombinase domain (cl02788) and all PLEs except PLE 3 encode predicted proteins with helix-turn-helix (HTH) DNA-binding domains (including those in the MarR family (COG1846) and general HTH superfamily members (cl21459)). PLE 2 encodes a protein with an InsE domain (COG2963), typical of a transposase or inactivated derivative. PLE 1 also encodes a protein with a domain found in the PSK transcription factor superfamily (cl01834) (Fig 1A).

In total, 51 out of the 208 *V*. *cholerae* isolates analyzed (~25%) harbor a PLE. PLE^+^ *V*. *cholerae* have been isolated between 1949–2011 (spanning the entire collection period in these studies [19,20]) from disparate locations including Egypt, Mozambique, Bangladesh and Thailand (S1 Table). PLEs are present in both classical and El Tor biotype strains, associated with the previous sixth and current seventh pandemics, respectively [23], with PLE 5 restricted to classical isolates and PLEs 1, 2, 3 and 4 present in El Tor strains. The temporal distribution of each PLE is such that previously prevalent PLEs disappear when new variants emerge (Fig 1B). All PLEs were located in chromosome II of *V*. *cholerae* (Fig 1C and S2 Table), and all but PLE 2 were integrated within the superintegron, a gene capture system with hundreds of gene cassettes of mostly unknown function [24].

### PLEs respond to and block infection by ICP1 phage

**Fig 2:**
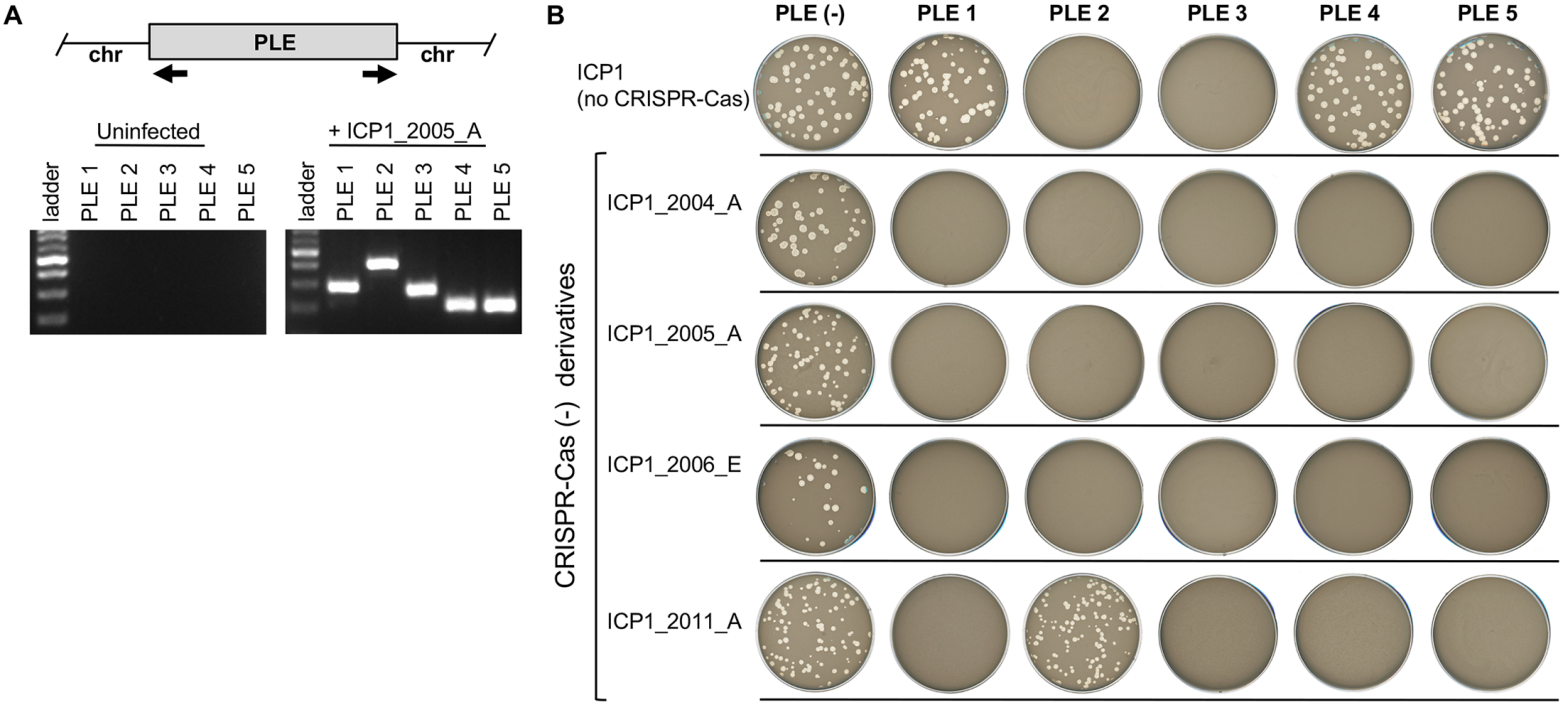
PLEs are induced by and protect against ICP1-related phages. (A) Agarose gel analysis of PCR products to detect circularized PLE following infection with ICP1_2005_A. The approximate locations of the primers used to detect circularized PLE (black arrows) are indicated on the schematic representation of a PLE integrated into chromosome II of *V*. *cholerae*. The resulting bands vary expectedly in size depending on the specific primer pair used to amplify the junction and all PCR products were confirmed by sequencing. (B) The sensitivity of each strain (top row) to different ICP1 isolates lacking CRISPR-Cas (left column) is shown. The efficiency of plaquing (which is the plaque count on the PLE^+^ host strain divided by that on the PLE^-^ host strain) is ~1 where plaques formed, and below the limit of detection (10^−8^) for phages that did not produce plaques.

PLE 1 was previously shown to excise upon phage ICP1_2011_A infection and block plaque formation by that phage [8]. Here, we evaluated the specificity of anti-phage activity for all five PLEs. We constructed PLE^+^ derivatives of *V*. *cholerae* E7946 (see Materials and methods) to compare PLE^+^/ PLE^-^ in the same strain background for these and all subsequent experiments. ICP1 isolates were assessed for their ability to form plaques on *V*. *cholerae* E7946 PLE^+^ derivatives. CRISPR-Cas^+^ ICP1 isolates were engineered to prevent CRISPR-mediated anti-PLE activity by deleting spacers in the CRISPR array or by deleting *cas2-3* [25], which possesses the nuclease activity required for target DNA degradation [10]. As shown in Fig 2A, all PLEs excised in response to ICP1 infection. Importantly, in the absence of ICP1 infection, PLE circularization was not detected (Fig 2A), nor could it be detected following infection with unrelated phages ICP2 or ICP3 (S1A Fig) or following treatment with mitomycin C (S1B Fig). For the mitomycin C treatment, we tested the PLE^+^ *V*. *cholerae* E7946 derivatives constructed in this study, as well as at least one PLE^+^ clinical isolate, since they may carry other prophages or mobile elements; however, we were unable to detect excised PLE in the absence of ICP1 infection. These results suggest that in contrast to SaPIs [11], resident prophages activated by the SOS response do not activate PLEs. Our data demonstrate that PLEs do not block all phages, but that they do block ICP1 phages; however, until the molecular determinants of PLE activity are deciphered, it remains possible that other phages not tested here may also stimulate PLEs in *V*. *cholerae*.

All PLEs blocked plaque formation by at least one ICP1 isolate (Fig 2B) and did not block plaque formation by ICP2 or ICP3 (S1C Fig), demonstrating that ICP1 interference is a conserved feature of these elements. The ability of ICP1 isolates to form plaques on a given PLE^+^ strain was an all or nothing phenotype: ICP1 isolates that formed plaques on a PLE^+^ host strain did so at the same efficiency as on a PLE^-^ strain, and when plaque formation was blocked, plaques could not be detected even when 10^8^ plaque forming units were added to a PLE^+^ host strain (Fig 2B). Interestingly, some ICP1 isolates recovered from cholera patient samples form plaques in the presence of certain PLEs independent of CRISPR activity (Fig 2B). This finding suggests that ICP1 isolates have evolved to prevent triggering PLE activity or that they have CRISPR-Cas independent mechanisms to perturb PLE activity once it has been triggered.

### PLE-mediated ICP1 interference abolishes phage production and is associated with a decrease in phage genome replication and accelerated cell lysis

**Fig 3:**
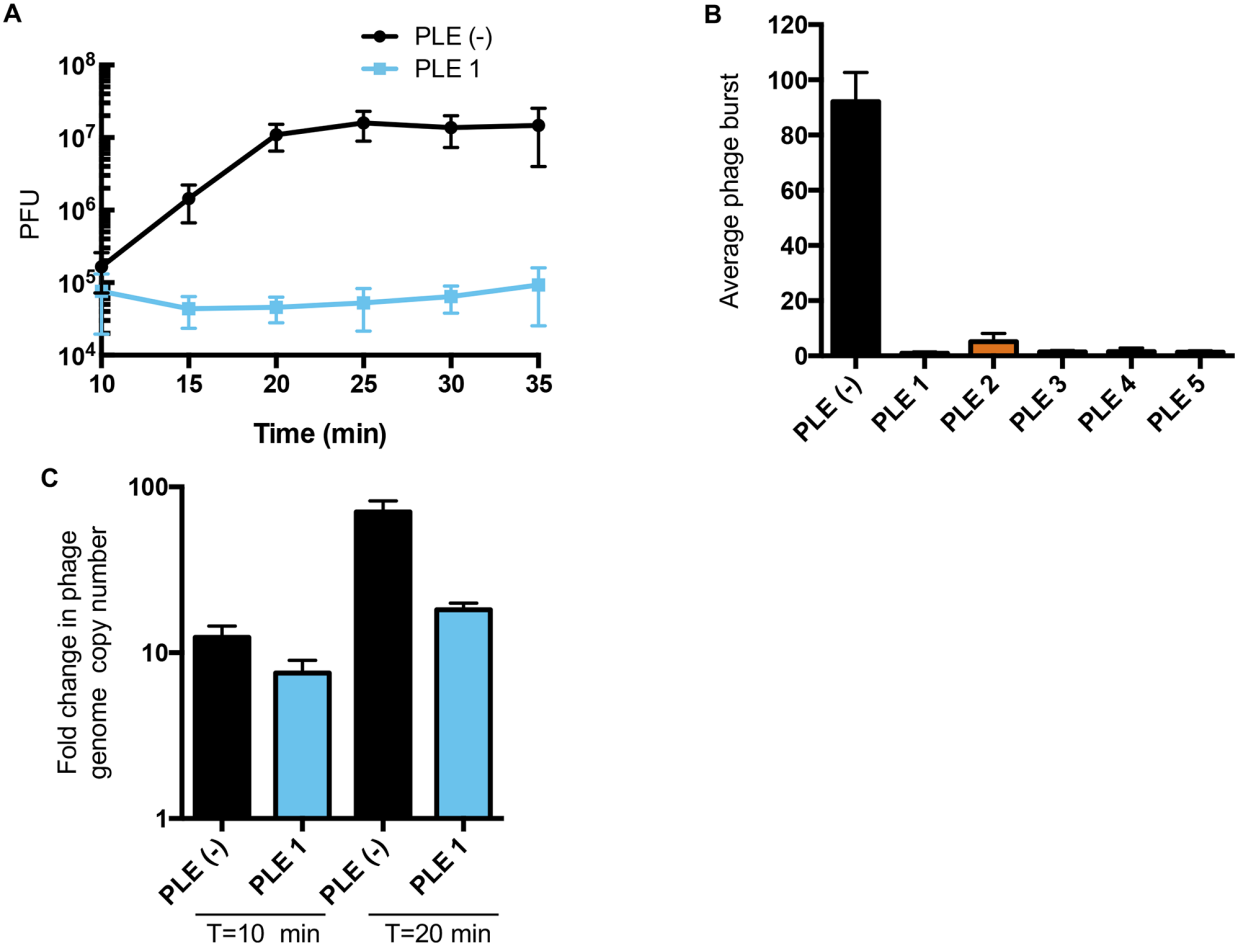
PLE-mediated ICP1 inhibition blocks phage burst and decreases phage genome replication. (A) One-step growth curve of phage ICP1_2006_E ΔCRISPR on *V*. *cholerae* +/- PLE 1. Starting PFU values (~10^5^) represent unabsorbed phage (<1%). These data, and one-step growth curves performed for the other PLEs, were used to calculate the average burst size of ICP1_2006_E ΔCRISPR on *V*. *cholerae* with or without the PLE indicated shown in (B). (C) Phage genome replication after infection of *V*. *cholerae* PLE 1^+/-^ with ICP1_2011_A ΔCRISPR as determined by qPCR. To determine fold change, samples 10 and 20 minutes post-infection were compared to the input sampled immediately after adding phage. For all panels, error bars indicate standard deviations of biological triplicates.

We quantified PLE-mediated ICP1 interference using one-step phage growth analysis. In a permissive *V*. *cholerae* PLE^-^ host, ICP1 infection culminated in the release of approximately 90 infectious virions per cell within 25 minutes (Fig 3A and 3B). Phage production was undetectable in PLE^+^ *V*. *cholerae* (Fig 3B). All ICP1 isolates use the O1 antigen receptor to initiate infection and the CRISPR-Cas^+^ wild-type phage isolates form plaques on all PLE^+^ strains (S2 Fig). Therefore, PLE activity does not block the phage genome from entering the cell, so we next quantified phage genome replication in the face of PLE activity. Interestingly, PLE 1 does not appear to perturb the kinetics of ICP1 replication in the first 10 minutes of infection, however, we found that PLE activity significantly reduces phage genome replication by approximately 4-fold by the end of the infection cycle (*p* < 0.005, Student’s *t*-test) (Fig 3C). Since phage genome replication is reduced but not eliminated, our results suggest that at least one additional mechanism of ICP1 interference is necessary to achieve the complete elimination of progeny virus production seen in the one-step phage growth analysis.

**Fig 4:**
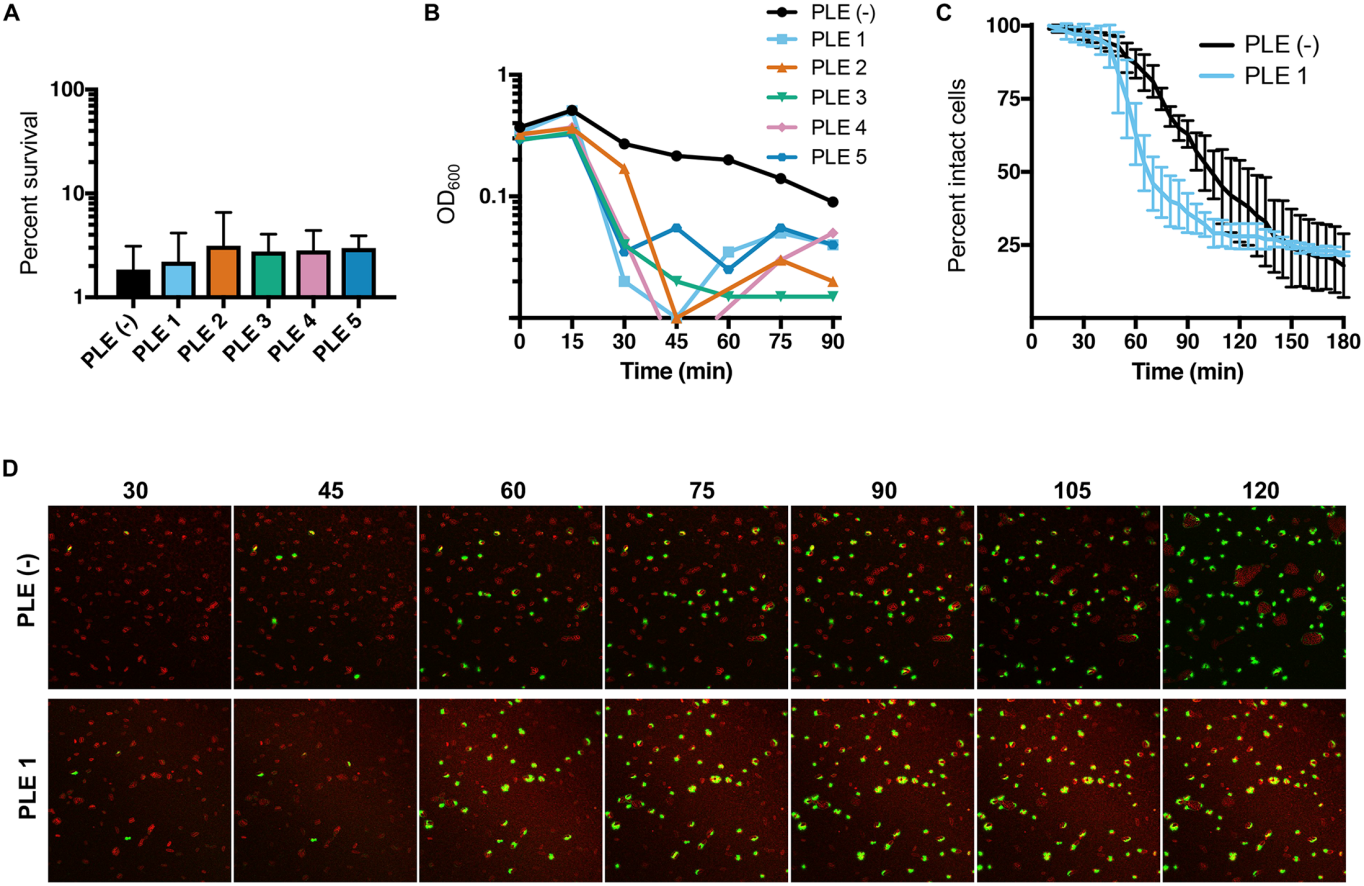
PLE induction results in accelerated cell lysis. (A) Survival of *V*. *cholerae* 15 minutes after infection with ICP1_2006_E ΔCRISPR at an MOI = 5. (B) OD_600_ values of phage-infected PLE^-^ versus PLE-containing strains of *V*. *cholerae*. Strains were grown to OD_600_ = 0.3 and then infected with ICP1_2006_E ΔCRISPR at an MOI = 5. Representative curves are based on results from three independent assays. (C) Cell lysis dynamics of phage-infected PLE^-^ versus PLE 1-containing strains of *V*. *cholerae* as determined by fluorescence microscopy following infection with ICP1_2006_E ΔCRISPR at an MOI = 5. Quantification of three independent biological replicates of (D), which show selected images of representative of PLE^-^ and PLE 1 *V*. *cholerae* infected with ICP1_2006_E ΔCRISPR over time. Samples were stained with the membrane stain FM 4–64 (red), and the DNA stain Sytox Green (green). For panels A and C, error bars indicate standard deviations of biological triplicates.

To investigate whether PLE activity protects phage infected *V*. *cholerae* cells from cell death, we quantified cell survival following infection with ICP1. Although PLEs block phage production, approximately equivalent levels of bacterial cell death were observed for PLE^+^ (97–98%) and PLE^-^ (98%) *V*. *cholerae* after infection (Fig 4A). In these analyses, we found that at a multiplicity of infection (MOI) of 5, PLE activity accelerates the lysis of *V*. *cholerae* following phage infection. Upon infection of PLE^-^ *V*. *cholerae*, we saw a slow increase in lysis of the bacterial culture as measured by OD_600_ (Fig 4B). In stark contrast, infection of the PLE^+^ strains resulted in both an accelerated decline and more complete clearance of the bacterial culture. Since monitoring of OD_600_ after phage infection for PLE^-^ at high MOI did not match the expected lysis timing obtained from one-step growth curves (Fig 3A), we performed time-lapse fluorescence microscopy to more precisely determine how PLE activity impacts bacterial cell lysis dynamics. In these experiments, we imaged PLE^+^ and PLE^-^ *V*. *cholerae* infected with phage at an MOI = 5 in the presence of the membrane stain FM 4–64 and the nucleic acid stain, Sytox Green, which brightly stains cells only when the membrane barrier is compromised. At the first several time points the two strains appeared identical, however, quantification of the loss of membrane integrity over time showed that cell lysis is accelerated in *V*. *cholerae* harboring PLE 1 compared to PLE^-^ (Fig 4C and 4D). Of note, the timing of the onset of lysis is the same in both strains, however, *V*. *cholerae* PLE 1 cells lysed in a more synchronized manner, and more PLE 1 cells were lysed at intermediate time points (for example, 40% percent of the PLE^+^ population lysed between 45–65 minutes post-infection, while 10% of PLE^-^ cells lysed during the same time period (Fig 4C)). The timing of lysis under the static conditions used for microscopy is delayed compared to in liquid culture (Fig 4B vs 4C), but collectively these results indicate that PLE activity results in accelerated cell lysis after phage infection. The mechanism of accelerated lysis, which could be mediated directly through a PLE-encoded product(s), through manipulation of the ICP1 lysis program, or even involve *V*. *cholerae* chromosomal product(s), and the relative contribution of PLE-mediated accelerated lysis to phage inhibition are not known.

### PLEs are mobilized by ICP1 infection

**Fig 5:**
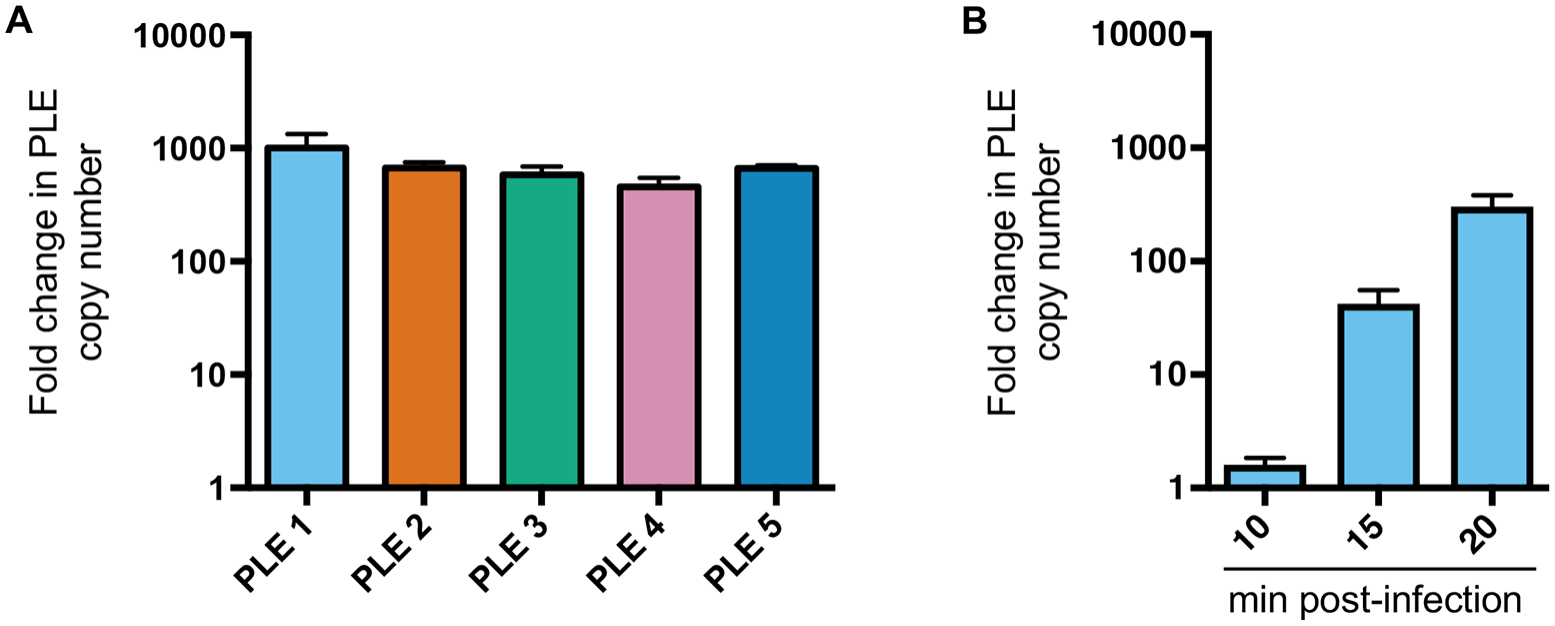
PLEs replicate following infection by ICP1-related phages. (A) PLE replication 20 minutes after infection with ICP1_2006_E ΔCRISPR as determined by qPCR. (B) PLE 1 replication was determined sequentially following infection with ICP1_2006_E ΔCRISPR as determined by qPCR. For both A and B, fold change was determined by comparing samples at the indicated time points to the input that was sampled immediately before adding phage. Error bars indicate standard deviations of biological triplicates.

**Fig 6:**
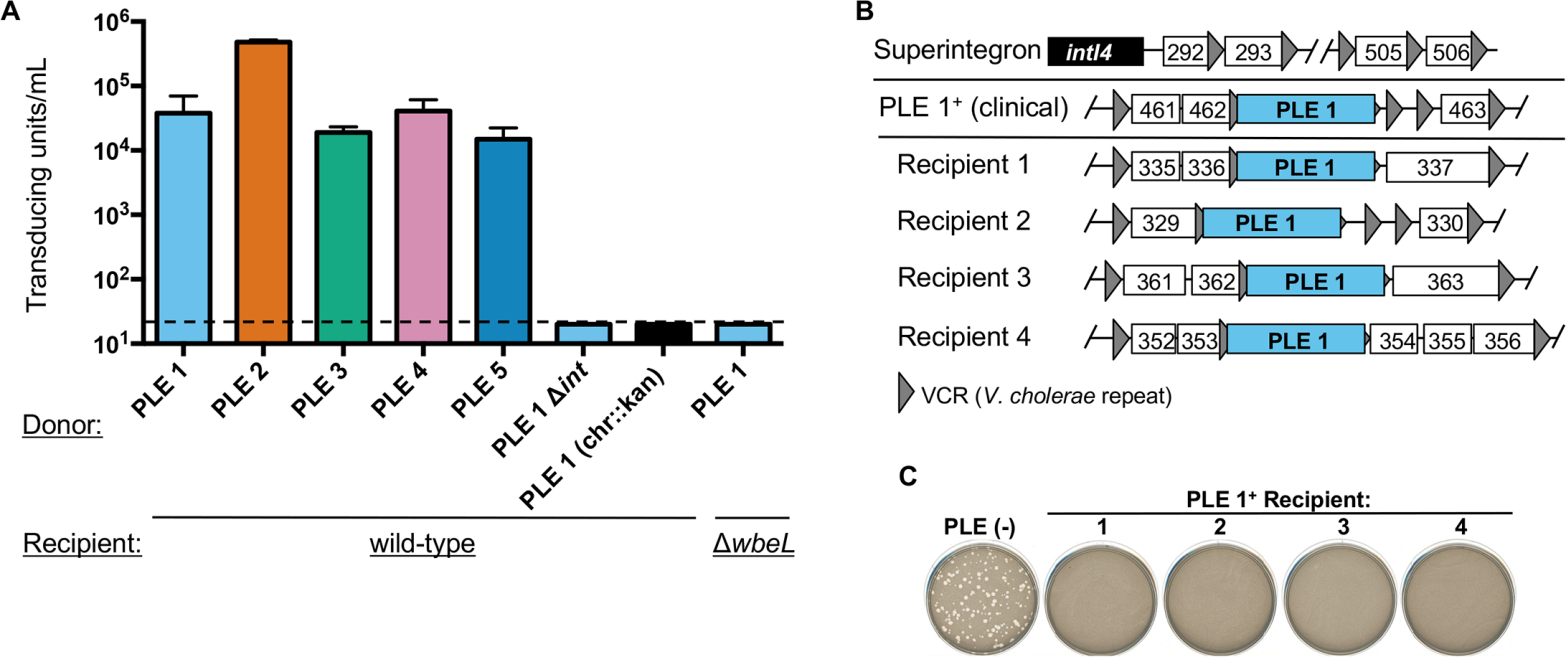
PLEs are mobilized following infection by ICP1-related phages. (A) PLE transducing units produced during infection with ICP1_2006_E ΔCRISPR. When the donor strain was a PLE variant harboring the kanamycin resistance cassette elsewhere in the chromosome (designated as chr::*kan*), no transduction could be detected, but we included only the PLE 1 variant for simplicity. The dashed line indicates the limit of detection for this assay. (B) PLE 1 integration into the *V*. *cholerae* superintegron. The *V*. *cholerae* superintegron is schematized in the top row: the superintegron integrase gene (*intI4)* with proximal and distal ORFs defining the superintegron boundaries are shown. ORFs are indicated by white boxes with 3 digit numbers corresponding to the VCA0XXX designation as observed in the N16961 reference genome. The position of PLE 1 in clinical isolates and four recipients generated by ICP1-mediated transduction is indicated by the PLE flanking genes within the superintegron. (C) Experimental PLE 1 transductants show resistance to ICP1 regardless of the position of PLE 1 within the superintegron. The sensitivity of each of the four PLE 1 recipients in (B) to ICP1_2011_A lacking CRISPR is shown. For panel A, error bars indicate standard deviations of biological triplicates.

Having established that PLE excision and cell lysis occurs in response to ICP1 infection, we next wanted to determine whether PLEs replicate and are packaged into infectious virions during ICP1 infection. We quantified PLE DNA before and after phage infection and observed that PLEs replicate to high copy number (Fig 5A). Sequential sampling of PLE 1 copy number after phage infection showed that PLE replication is low 10 minutes post-infection, but increases substantially 15 and 20 minutes post-infection (Fig 5B), which may indicate a switch from ICP1 replication (which occurs unperturbed early in infection (Fig 3C)) to PLE replication in infected cells. After replication, SaPI DNA is packaged into infectious phage-like transducing particles composed exclusively of helper phage virion proteins [14,15]; on entry into new cells, SaPI DNA integrates in a site-specific manner into the chromosome [11]. To investigate whether PLEs are similarly mobilized by ICP1 infection, we inserted a kanamycin resistance marker downstream of the last ORF in each PLE and measured PLE transduction frequency with ICP1. We first confirmed that introduction of the kanamycin resistance cassette did not alter PLE replication (S3 Fig). We then added cell-free supernatants from ICP1-infected PLE::*kan* cultures to recipient *V*. *cholerae* (Δ*lacZ*:: spec) and plated on agar plates supplemented with both antibiotics to select for cells that acquired PLE. PLE transducing units were detected at a frequency of ~10^4^−10^5^ per 10^8^ infected cells, indicating that the overall efficiency of PLE packaging into infectious virions is low (fewer than 1 transducing unit produced per 100 infected cells [PLE 2] or per 1000 infected cells [other PLEs] (Fig 6A)). As a control, when the same marker was inserted elsewhere in the chromosome of PLE^+^ strains (shown as PLE 1 (chr::*kan*)), transduction was below the limit of detection, indicating that the packaging of PLE is not random (Fig 6A). We hypothesized that PLE transduction would be dependent on the PLE encoded integrase. To test this, we constructed a PLE 1 Δ*int* mutant and found that transduction was below the limit of detection, consistent with its predicted role in mediating PLE integration in recipient cells. To begin to address if PLEs are packaged into particles composed of ICP1 proteins, we evaluated if PLE transduction requires the *V*. *cholerae* lipopolysaccharide O1 antigen (which is the ICP1 receptor [3]). Indeed, we found that PLE 1 could not be transduced to O1-antigen deficient *V*. *cholerae* (Δ*wbeL*) (Fig 6A). Our results show that PLE transduction has the same receptor requirements as ICP1 infection and are consistent with the hypothesis that PLE DNA is packaged into virions composed of ICP1 proteins, although further analysis is required to evaluate the molecular nature of PLE transduction.

We determined the site of PLE integration in recipient cells by amplifying and sequencing PLE chromosomal junctions with arbitrary primed PCR. For all PLEs, integration occurred in a site-specific manner (Fig 6B and S3 Table). PLEs 1, 3, 4 and 5 integrated into a *V*. *cholerae* repeat (VCR). VCRs are ~124 bp sequences found flanking gene cassettes in the *V*. *cholerae* superintegron [26]. VCRs are present in >100 copies, therefore ICP1-mediated PLE transduction yielded recipients in which the PLE integrated into a VCR and was consequently surrounded by unique flanking genes (Fig 6B). PLE transductants showed phenotypic conversion to ICP1 resistance (Figs 2B and 6C), and the position of the newly acquired PLE within the superintegron did not appear to impact phenotypic conversion to ICP1 resistance (Fig 6C). In contrast to the other PLEs, PLE 2 integrated into VCA0581 (encoding a hypothetical protein), a finding that is consistent with the observation that of the PLE encoded integrases, the PLE 2 integrase is the most divergent (Fig 1A). We also determined the site of PLE integration in naturally occurring PLE^+^ *V*. *cholerae* isolates and found that the site of integration was the same as in experimental transductants (that is, PLE 2 integrated into VCA0581 and PLEs 1, 3, 4 and 5 were found integrated in a VCR (S2 Table)). For natural *V*. *cholerae* isolates harboring PLEs integrated within a VCR, all PLE^+^ *V*. *cholerae* isolates were identical with respect to PLE flanking genes, indicating vertical transmission of PLEs in nature. Therefore, we found no evidence of ICP1-mediated PLE transduction (horizontal acquisition) in the natural strains we tested (S2 Table), however it is possible that those strains are not representative of the breadth of PLE^+^ *V*. *cholerae* in nature.

CRISPR activity is necessary for phage ICP1_2011_A replication on a PLE 1 host [8], but unexpectedly, we found that PLE 1 transduction efficiency was unchanged when CRISPR was active (S4 Fig). This indicates that the extent to which PLEs are packaged, potentially in ICP1 structural components, is not responsible for ICP1 interference. Such a result also implies that PLE DNA copy number is not the component that limits PLE transduction, but potentially that phage components required for packaging PLE particles may be limited during PLE-mediated phage inhibition.

## Discussion

We have shown that PLEs are persistent genomic islands in geographically disparate *V*. *cholerae* isolates that provide highly efficient protection from a predatory phage. Our data demonstrate that all PLEs provide protection from ICP1, the dominant *V*. *cholerae* phage found in cholera patient stool samples from Bangladesh [3]. The dominance of this phage and our current finding of a dedicated ICP1-defense system in *V*. *cholerae* isolates collected over a 60-year sampling period serve to further validate that interactions with ICP1-related phages have been a significant driver in the long-term evolution and selection of *V*. *cholerae*. Accessory genetic elements, like the PLEs, confer a fitness advantage in the face of ICP1 predation without the costs associated with compromising core functions through mutation. PLEs have no sequence similarity to other known anti-phage systems, and thus bioinformatic-based predictions to understand how PLEs block phage replication are largely uninformative. PLEs do, however, show some functional similarities to SaPIs, which are well known for their ability to parasitize helper phages to permit their own packaging and spread. We have provided evidence that like SaPIs, PLE transmission is facilitated by phage infection, and we have identified features of the PLE life cycle that provide insight into understanding how these evolutionarily conserved elements function.

In contrast to SaPIs, PLEs do not encode identifiable regulatory, replication or packaging modules [12]. Nonetheless, our data demonstrate that ICP1 infection of PLE^+^ *V*. *cholerae* leads to PLE excision, replication and packaging. PLE activity is characterized by accelerated cell lysis (Fig 4B) and a complete block in progeny phage production (Fig 3B), phenotypes that to our knowledge have not been reported for SaPIs. PLE activity is therefore similar to abortive infection systems, which act at the expense of the infected cell to eliminate phage production and protect the surrounding clonal population from infection. Although PLEs do capitalize on ICP1 infection to spread to neighboring cells, it appears to be relatively inefficient, raising the possibility that PLEs are ancient phage parasites that have evolved into specialized phage defense systems at the cost of their own horizontal transfer. In support of this idea, we found that PLE packaging following ICP1 infection is not responsible for ICP1 interference, since PLE transduction still occurs when the phage’s CRISPR-Cas system is active and PLEs are not inhibitory (S4 Fig). The robust anti-phage activity of PLEs may be mediated in part by accelerated host cell lysis. Since cell lysis is not premature for all infected cells per se, we do not expect that PLE-mediated accelerated lysis is sufficient to explain the complete block in phage production. However, even slight deviations from the precisely controlled expression of the genetic information needed to amplify the phage genome, assemble viral particles and package phage DNA could have dramatic effects on phage viability, and there are likely some PLE^+^ cells in which the phage’s developmental program is incomplete prior to lysis. Similarly, PLE transduction may be limited by accelerated host cell lysis if phage components required for particle formation do not reach optimal levels prior to lysis. In addition, PLE activity interferes with phage genome replication, which may act in concert with accelerated host cell lysis and/or other yet to be identified mechanisms to efficiently block phage production. As a phage parasite, PLE packaging is likely completely dependent on phage-encoded structural proteins, and thus favoring accelerated cell lysis may come at a cost for the PLE. Since the requirements of PLE-mediated accelerated cell lysis have not been elucidated, it remains to be seen whether relieving accelerated cell lysis both restores some progeny phage production and enhances PLE transduction, as our model would predict.

The evolution of a phage-encoded CRISPR-Cas system [8] to overcome PLE activity is remarkable and may speak to the relative strength of PLEs as defensive barriers in comparison to SaPIs. Some SaPIs decrease phage titer by only ~3x and still prevent plaque formation [16], however, PLEs eliminate progeny phage production entirely. The mechanisms allowing phage to coevolve and overcome these genomic islands also differ. Helper phages that fail to induce SaPI activity can be readily selected for under laboratory conditions because SaPI induction depends on a single, dispensable helper phage-encoded protein [27,28]. Characterization of such mutants has led to the identification of SaPI inducing proteins, which function as phage-encoded antirepressors that induce SaPI excision, replication and packaging. In contrast to the SaPI-helper phage paradigm, we have been unable to use experimental evolution experiments to select for ICP1 mutants that escape PLE-mediated interference. This indicates that there may be an insurmountable fitness cost to altering or losing the PLE inducing cue and/or that multiple ICP1 products induce PLE activity to permit redundancy and ensure an adequate response by the PLE^+^ host. By deleting CRISPR-Cas in our collection of ICP1 isolates, we have identified certain phage isolates that can escape PLE mediated interference, highlighting the need to study naturally evolved bacterial and viral populations. ICP1 isolates differ by thousands of single nucleotide polymorphisms and by the presence of accessory modules like CRISPR-Cas [3,8], making bioinformatic approaches to identify the defining feature(s) mediating PLE escape ineffective. As we strive towards a more comprehensive understanding of the role of phage in shaping bacterial communities in health and disease, it is imperative that we consider the vast gene pool enabling the acquisition of novel traits and continued coevolution that inherently cannot be replicated in laboratory evolution experiments. The long-term interactions between *V*. *cholerae* and ICP1 serve as a useful paradigm to understand the evolution of phage-resistance and counter-resistance in the context of human disease, and may allow for the potential manipulation of these systems for therapeutic or prophylactic benefit.

## Materials and methods

### Strains and growth conditions

Strains utilized in this study are listed in S4 Table. PLEs were transduced into *V*. *cholerae* E7946 [29] (described below) to generate PLE^+^ derivatives in the same strain background for comparisons in these studies. Bacteria were routinely grown at 37°C on lysogeny broth (LB) agar or in LB broth with aeration. Media was supplemented with kanamycin (75 μg/ml), spectinomycin (100 μg/ml), and/or streptomycin (100 μg/ml) when appropriate.

### Generating mutant strains

Antibiotic resistance markers were introduced into *V*. *cholerae* strains by natural transformation as described [30]. Splicing by overlap extension (SOE) PCR was used to generate all PCR constructs. Primer sequences are available upon request. In order to generate PLE^+^ derivatives in the same strain background, PLEs were marked with a kanamycin resistance cassette downstream of the last ORF. PLEs 1–3 were mobilized by transduction with an ICP1 isolate into *V*. *cholerae* E7946. Natural transformation and transduction were used to generate *V*. *cholerae* E7946 harboring PLE 4 or PLE5 in the following manner: *V*. *cholerae* E7946 was made competent by growth on chitin [30] and ~2μg purified genomic DNA from the kanamycin resistant PLE 4 or PLE 5 derivative strain was added and the mixture was incubated at 30°C overnight and then plated onto LB kanamycin plates. Kanamycin resistant colonies were screened by PCR to ensure the desired incorporation of the entire PLE, and then to ensure a clean genetic background, these derivatives were used as donors in ICP1_2011_A-mediated transduction assays into *V*. *cholerae* E7946. For all PLE^+^ strains, the kanamycin resistance cassette was removed using cotransformation [31] of the wild-type locus with a selected product to replace *lacZ* with a spectinomycin resistance marker. The spectinomycin resistance marker was subsequently replaced by the wild-type *lacZ* locus and screening for desired transformants on plates containing 40 μg/mL 5- bromo-4-chloro-3-indolyl-β-_D_-galactopyranoside. The PLE 1 integrase deletion construct was constructed using FLP-FRT recombination as described [32]. Mutations in ICP1-related phages were generated using CRISPR-Cas mediated genome engineering as described [25].

### PLE circularization

*V*. *cholerae* E7946 PLE^+^ were grown to OD_600_ = 0.3 and infected with phage at an MOI of 5. Samples were taken 20 minutes post-infection, boiled and used as template for PCR to detect the circularized PLE using outward facing primers as depicted in Fig 2A. In order to determine if PLEs circularize in response to ICP2 or ICP3 [3], boiled plaques on *V*. *cholerae* E7946 PLE^+^ served as a template for circularization PCR. Positive controls using plaques on PLE^+^ strains infected with ICP1-related phages were used in all assays. In order to test if induction of the SOS response could stimulate PLE circularization, PLE^+^ strains (both *V*. *cholerae* E7946 PLE^+^ transductants and clinical isolates naturally found to harbor each PLE) were grown to OD_600_ = 0.3 and treated with mitomycin C (at 20 ng/mL and 100 ng/mL) for 30 minutes. Treated samples were boiled and used as a template for PCR as above. All PCR reactions were carried out under identical conditions for 30 cycles with positive controls in all assays. Circularization products were confirmed by sequencing.

### Phage infection

Phage susceptibility was determined using the soft agar overlay method as described [25]. One-step growth curves were used to determine the average phage burst size [33]. One-step growth curves were performed in triplicate and the phage burst is reported as the means ± SD (Standard Deviation) in Fig 3B. Bacterial survival was determined following infection of *V*. *cholerae* E7946 and its PLE^+^ derivatives with phage as follows: strains were grown to an OD_600_ = 0.3 and infected with ICP1_2006_E ΔCRISPR (MOI = 5). After 15 minutes of incubation at 37°C with aeration, serial dilutions of each infected culture were plated on LB streptomycin plates. Uninfected cultures were plated for CFUs immediately prior to infection and the percent survival was calculated as (CFU_(phage treatment)_/CFU_(uninfected)_) x100. The average percent survival was determined from three biological replicates and is reported as the means ± SD in Fig 4A. The kinetics of phage infection of *V*. *cholerae* E7946 and its PLE^+^ derivatives with ICP1_2006_E ΔCRISPR were performed at the MOI indicated at 37°C with aeration.

### Fluorescence microscopy

*V*. *cholerae* strains were grown to an OD_600_ = 0.3 and then concentrated 5-fold before being infected with ICP1_2006_E ΔCRISPR (MOI = 5) in a 200 μL volume. 1 μL each of 0.05mM Sytox Green nucleic acid stain (Thermo Fisher Scientific) and 1 μg/μL FM 4–64 (Thermo Fisher Scientific) were added and the mixture was incubated for 5 minutes at room temperature. 10 μL of the cell suspension was then placed on an agarose pad (1.5% diluted in LB) made using a gene frame seal (Thermo Scientific). Images were taken at 5-min intervals with the stage set to 37°C with an Olympus FV1000 confocal microscope with a 60X objective. The average percent intact cells were determined from three biological replicates and are reported as the means ± SD in Fig 4C.

### Transduction experiments

For transduction assays, phage (MOI = 5) were added to *V*. *cholerae* strains at an OD_600_ = 0.3 for 5 minutes at 37°C with aeration. The mixture was centrifuged and washed to remove unabsorbed phage, resuspended in fresh LB broth and incubated for 30 minutes at 37°C with aeration. The lysate was treated with chloroform and centrifuged to remove bacterial debris. 100 μL lysate was mixed with 100 μL overnight culture of recipient *V*. *cholerae* (Δ*lacZ*:: spec as wild-type recipient, or Δ*wbeL* Δ*lacZ*:: spec as indicated) at 37°C for 1 hour. This mixture was plated on LB agar plates supplemented with kanamycin and spectinomycin to enumerate transducing units. PLE transducing units were calculated from three biological replicates and are reported as the means ± SD of each donor/recipient pair indicated in Fig 6A. A kanamycin cassette inserted into the neutral gene VC1807 served as donor strains for detecting transduction of non-PLE associated sequence from PLE^+^ strains. The site of PLE integration in clinical isolates and experimental transductants was determined by arbitrary-primed PCR [34].

### Real-time quantitative PCR

qPCR reactions were performed with iQ SYBR Green Supermix (Bio-Rad) using a CFX Connect Real-Time PCR Detection system (Bio-Rad). For all assays, at least three independent samples were tested for each condition and each template sample was tested in technical duplicate. In order to quantify phage genome replication, *V*. *cholerae* was grown to OD600 = 0.3. Phage (at an MOI = 0.1) were added and incubated at 37°C with aeration. At the times indicated, 20 μL samples were taken, boiled and diluted 1:50 and used as template for qPCR, which was compared to the input sampled immediately after adding phage. Phage-specific primers zac68 (5’-CTGAATCGCCCTACCCGTAC-3’) and zac69 (5’-GTGAACCAACCTTTGTCGCC-3’) were used in this analysis. For PLE replication following phage infection *V*. *cholerae* was grown to OD600 = 0.3. Phage (at an MOI = 5) were added and incubated at 37°C with aeration. Samples were taken as above, boiled and diluted 1:1000 and used as template for qPCR for comparison to the input that was sampled immediately before adding phage. Primers universal for all PLEs were used for qPCR: zac14 (5’-AGGGTTTGAGTGCGATTACG-3’) and zac15 (5’-TGAGGTTTTACCACCTTTTGC-3’).

## Supporting information

S1 Fig PLEs do not circularize following infection by ICP2 or ICP3 or following treatment with mitomycin C and do not protect against ICP2 or ICP3 infection. (A) Agarose gel analysis of PCR products to detect circularized PLE following infection with ICP1_2005_A, ICP2 or ICP3. The approximate locations of the primers used to detect circularized PLE (black arrows) are indicated on the schematic representation of a PLE integrated into chromosome II of *V*. *cholerae*. The resulting bands vary expectedly in size depending on the specific primer pair used to amplify the junction. (B) Agarose gel analysis of PCR products to detect circularized PLE following treatment with mitomycin C. E7946 PLE^+^ derivatives (E7946^+^) and a clinical isolate harboring each PLE are indicated. (C) The sensitivity of each strain (top row) to different phage (left column) is shown. The efficiency of plaquing (which is the plaque count on the PLE^+^ host strain divided by that on the PLE^-^ host strain) is ~1 where plaques formed.
(TIF)

S2 Fig CRISPR-Cas^+^ ICP1 phage isolates form plaques on PLE^+^ *V*. *cholerae*. The sensitivity of each strain (top row) to different CRISPR-Cas^+^ phage (left column) is shown. The efficiency of plaquing (which is the plaque count on the PLE^+^ host strain divided by that on the PLE^-^ host strain) is ~1 where plaques formed.
(TIF)

S3 Fig PLEs marked with a kanamycin resistance cassette for transduction experiments replicate in response to phage infection. PLE replication 20 minutes after infection with ICP1_2006_E ΔCRISPR as determined by qPCR.
(TIF)

S4 Fig PLE transduction is not eliminated by ICP1 CRISPR. PLE 1 transducing units produced during infection with ICP1_2011_A, the CRISPR (-) derivative of this phage does not have a PLE-directed spacer [8]. Error bars indicate standard deviations of biological triplicates.
(TIF)

S1 Table The geographic and temporal origin of PLE containing *V*. *cholerae*. (PDF)

S2 Table PLE integration sites in naturally occurring *V*. *cholerae* isolates. (PDF)

S3 Table PLE integration sites in experimental ICP1-mediated PLE transduction experiments. (PDF)

S4 Table Bacterial strains, plasmids and phages used in this study. (PDF)

S1 Dataset Protein sequences for all predicted PLE-encoded ORFs. (RTF)
