# Supplementary figures and images for: A highly specific phage defense system is a conserved feature of the Vibrio cholerae mobilome
Source: PLoS Genet. 2017 Jun 8;13(6):e1006838. doi: 10.1371/journal.pgen.1006838 (PMC5481146; doi:10.1371/journal.pgen.1006838)

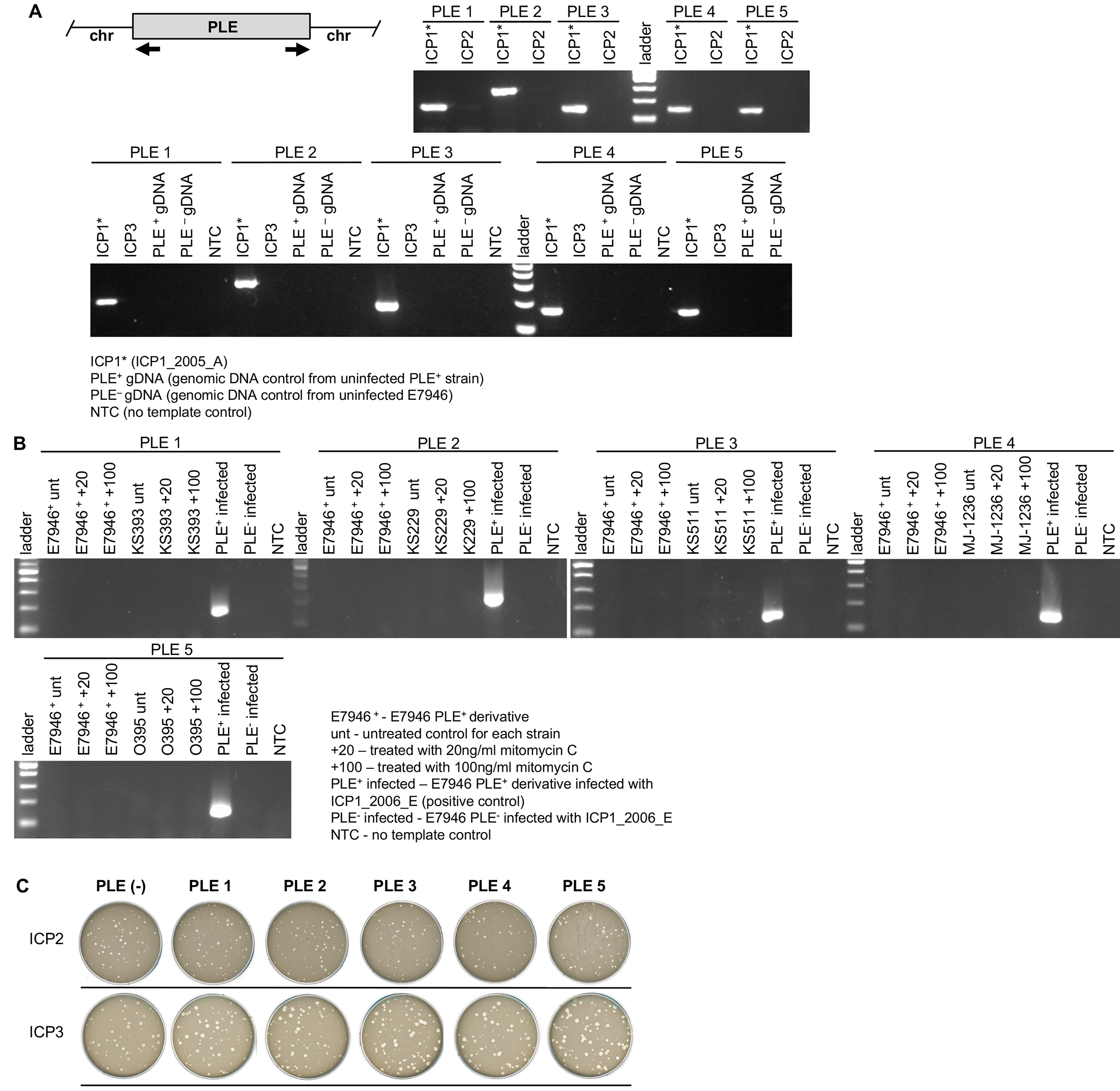

Supplement: S1 Fig — (A) Agarose gel analysis of PCR products to detect circularized PLE following infection with ICP1_2005_A, ICP2 or ICP3. The approximate locations of the primers used to detect circularized PLE (black arrows) are indicated on the schematic representation of a PLE integrated into chromosome II of V. cholerae. The resulting bands vary expectedly in size depending on the specific primer pair used to amplify the junction. (B) Agarose gel analysis of PCR products to detect circularized PLE following treatment with mitomycin C. E7946 PLE+ derivatives (E7946+) and a clinical isolate harboring each PLE are indicated. (C) The sensitivity of each strain (top row) to different phage (left column) is shown. The efficiency of plaquing (which is the plaque count on the PLE+ host strain divided by that on the PLE- host strain) is ~1 where plaques formed. (TIF) [file pgen.1006838.s001.tif]

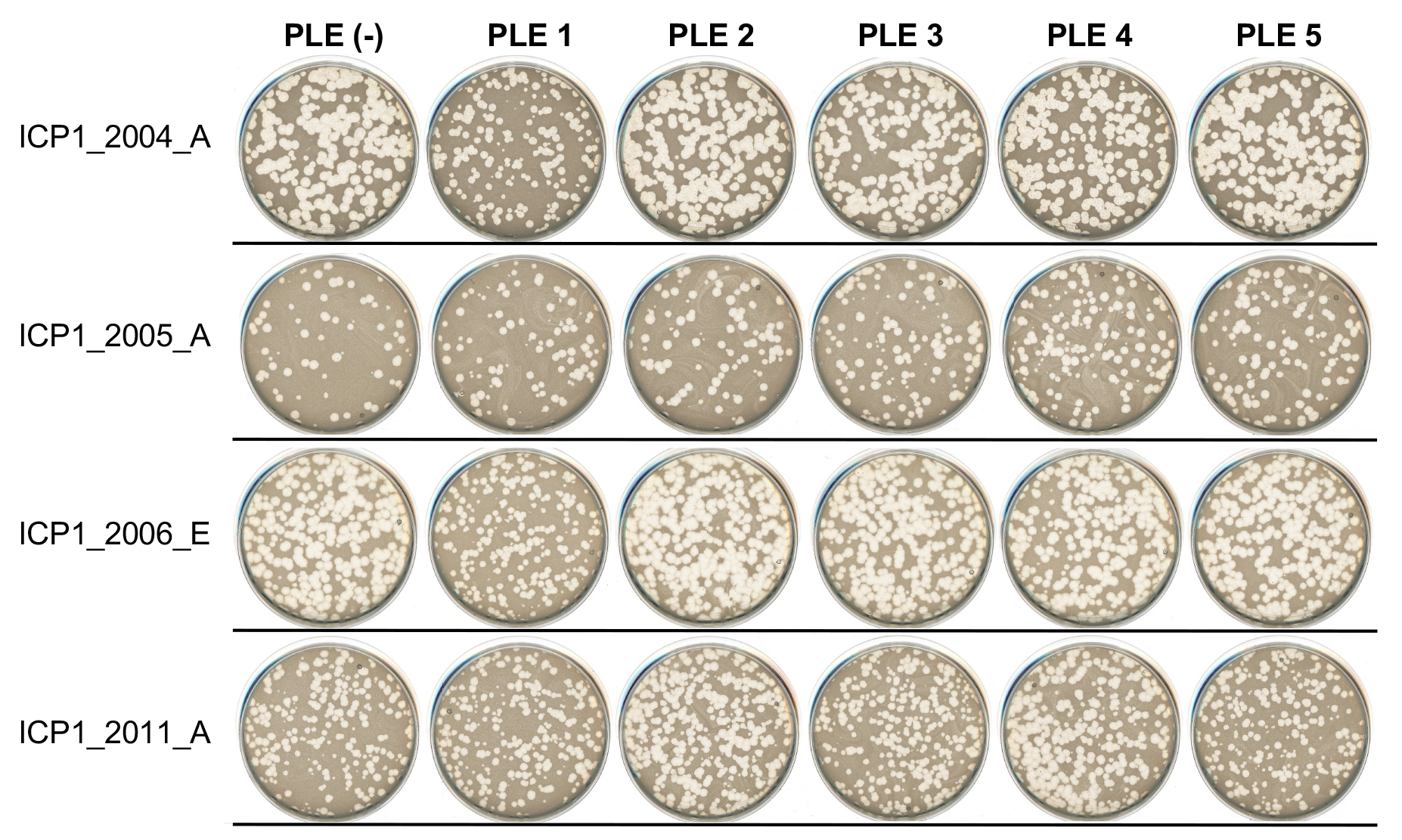

Supplement: S2 Fig — The sensitivity of each strain (top row) to different CRISPR-Cas+ phage (left column) is shown. The efficiency of plaquing (which is the plaque count on the PLE+ host strain divided by that on the PLE- host strain) is ~1 where plaques formed. (TIF) [file pgen.1006838.s002.tif]

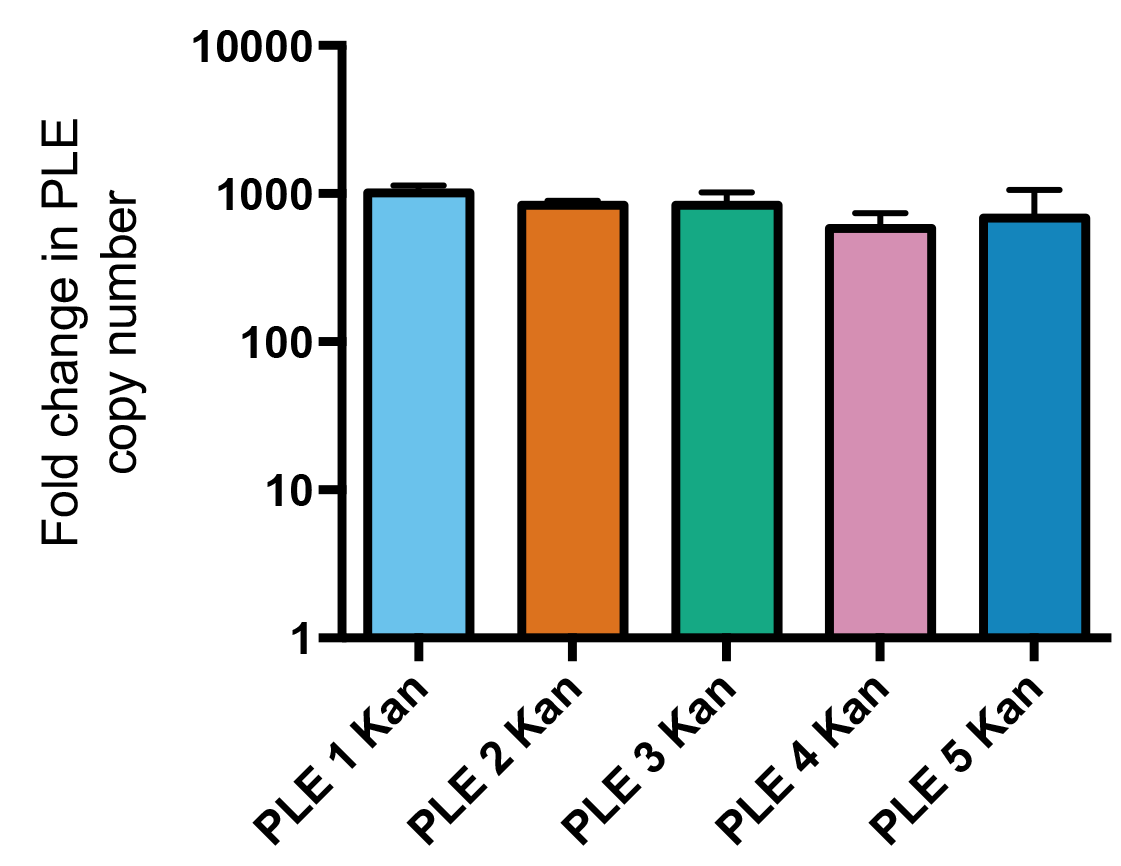

Supplement: S3 Fig — PLE replication 20 minutes after infection with ICP1_2006_E ΔCRISPR as determined by qPCR. (TIF) [file pgen.1006838.s003.tif]

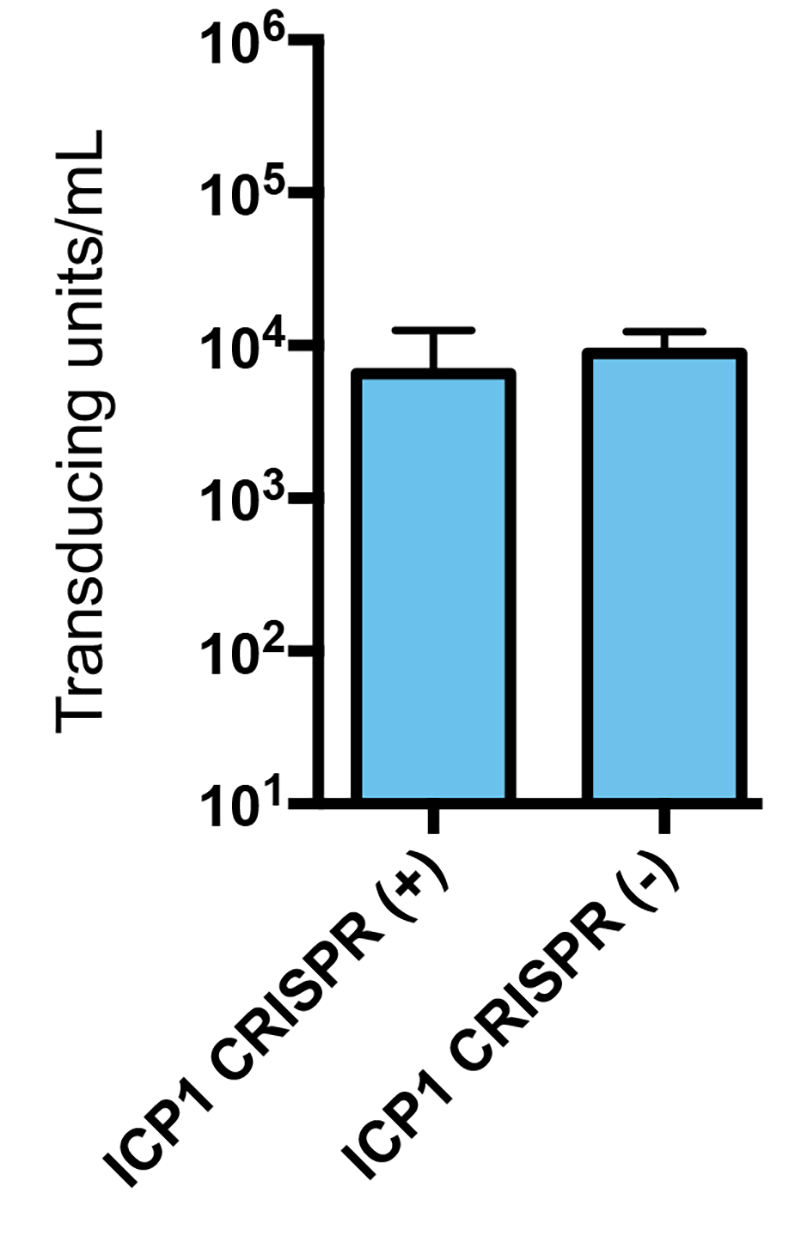

Supplement: S4 Fig — PLE 1 transducing units produced during infection with ICP1_2011_A, the CRISPR (-) derivative of this phage does not have a PLE-directed spacer [8]. Error bars indicate standard deviations of biological triplicates. (TIF) [file pgen.1006838.s004.tif]
