# Supplementary material for: A highly specific phage defense system is a conserved feature of the Vibrio cholerae mobilome
Source: PLoS Genet. 2017 Jun 8;13(6):e1006838. doi: 10.1371/journal.pgen.1006838 (PMC5481146; doi:10.1371/journal.pgen.1006838)
Supplement: S1 Table — (PDF) [file pgen.1006838.s005.pdf]

S1 Table. The geographic and temporal origin of PLE containing *V. cholerae*

PLE[a]	Strain[b]	Isolation Place[c]	Isolation Year
PLE 1	KS510	Bangladesh (ICDDR,B)	2009
	KS409	Bangladesh (ICDDR,B)	2010
	KS393	Bangladesh (ICDDR,B)	2011
	KS396	Bangladesh (ICDDR,B)	2011
	KS398	Bangladesh (ICDDR,B)	2011
	KS400	Bangladesh (ICDDR,B)	2011
	KS401	Bangladesh (ICDDR,B)	2011
PLE 2	KS344	Bangladesh (ICDDR,B)	2005
	4642	India	2006
	4656	India	2006
	KS229	Bangladesh (ICDDR,B)	2006
	KS255	Bangladesh (ICDDR,B)	2006
	4646	India	2007
	KS348	Bangladesh (ICDDR,B)	2007
	KS517	Bangladesh (ICDDR,B)	2008
PLE 3	KS511	Bangladesh (ICDDR,B)	2008
	KS516	Bangladesh (ICDDR,B)	2009
	KS515	Bangladesh (ICDDR,B)	2009
	KS39	Bangladesh (ICDDR,B)	2009
PLE 4	MJ-1236	Bangladesh (M)	1994
	A346(1)	Bangladesh	1994
	MJ1485	Bangladesh (M)	1994
	A346(2)	Bangladesh	1994
	B33	Mozambique	2004
	1362	Mozambique	2005
	1346	Mozambique	2005
	1627	Mozambique	2005
PLE 5	A51	Egypt	1949
	A68	Egypt	1949
	A60	Thailand	1958
	A49	NI	1962
	A66	Bangladesh	1962
	A50	Bangladesh	1963
	A46	NI	1964
	O395	India	1965
	A70	Bangladesh	1969
	GP8	India	1970
	A59	India	1970
	A61	India	1970
	GP16	India	1971
	A57	India	1980
	A76	Bangladesh	1982
	A389	Bangladesh (M)	1987
	A103	NI	1990
	A111	NI	1990
	A279	Sweden	1990
PLE (unknown)	A390	Bangladesh (M)	1987
	A241	Vietnam	1989
	A245	Vietnam	1989
	4110	Vietnam	1995
	4121	Vietnam	2004

[a] PLE designation: based on BLASTN analysis of whole genome sequence data [19, 20]. For PLE (unknown), the whole genome sequence data generated by Mutreja *et al.*, [19] was not sufficient to adequately analyze PLEs from these isolates and the corresponding strains were unavailable for phenotypic analysis or re-sequencing.

[b] Strain name, isolation date and place is from Mutreja *et al.*, [19] or (for KS strains) from Dalia *et al.*, [20].

[c] For locations: NI = No information; Additional details regarding isolation of strains from Bangladesh if available are indicated as (M) = Matlab, (ICDDR,B) = ICDDR,B located in Dhaka.
