# Supplementary material for: A highly specific phage defense system is a conserved feature of the Vibrio cholerae mobilome
Source: PLoS Genet. 2017 Jun 8;13(6):e1006838. doi: 10.1371/journal.pgen.1006838 (PMC5481146; doi:10.1371/journal.pgen.1006838)
Supplement: S2 Table — (PDF) [file pgen.1006838.s006.pdf]

S2 Table. PLE integration sites in naturally occurring *V. cholerae* isolates

PLE	Strain[a]	Integration Information
PLE 1	KS393 (ICDDR,B 2011)	In VCR between VCA0462 and VCA0463
	KS510 (ICDDR,B 2009)	In VCR between VCA0462 and VCA0463
	KS409 (ICDDR,B 2010)	In VCR between VCA0462 and VCA0463
	KS396 (ICDDR,B 2011)	In VCR between VCA0462 and VCA0463
	KS398 (ICDDR,B 2011)	In VCR between VCA0462 and VCA0463
	KS400 (ICDDR,B 2011)	In VCR between VCA0462 and VCA0463
	KS401 (ICDDR,B 2011)	In VCR between VCA0462 and VCA0463
PLE 2	KS229 (ICDDR,B 2006)	Interrupting VCA0581
	KS344 (ICDDR,B 2005)	Interrupting VCA0581
	KS255 (ICDDR,B 2006)	Interrupting VCA0581
	KS348 (ICDDR,B 2007)	Interrupting VCA0581
	KS517 (ICDDR,B 2008)	Interrupting VCA0581
PLE 3	KS511 (ICDDR,B 2008)	In VCR between VCA0441 and VCA0440
	KS516 (ICDDR,B 2009)	In VCR between VCA0441 and VCA0440
	KS515 (ICDDR,B 2009)	In VCR between VCA0441 and VCA0440
	KS39 (ICDDR,B 2009)	In VCR between VCA0441 and VCA0440
PLE 4	MJ-1236 (Bangladesh, 1994)	In VCR between VCA0491 and VCA0492
	B33 (Mozambique, 2004)	In VCR between VCA0491 and VCA0492
PLE 5	O395 (India 1965)	In VCR between VCA0481 and VCA0480
	A50 (Bangladesh 1963)	In VCR between VCA0481 and VCA0480
	A57 (India 1980)	In VCR between VCA0481 and VCA0480
	A68 (Egypt 1949)	In VCR between VCA0481 and VCA0480
	A111 (N.I 1990)	In VCR between VCA0481 and VCA0480

[a] Strain number is shown beside location and year of isolation
