# Supplementary material for: A highly specific phage defense system is a conserved feature of the Vibrio cholerae mobilome
Source: PLoS Genet. 2017 Jun 8;13(6):e1006838. doi: 10.1371/journal.pgen.1006838 (PMC5481146; doi:10.1371/journal.pgen.1006838)
Supplement: S3 Table — (PDF) [file pgen.1006838.s007.pdf]

S3 Table. PLE integration sites in experimental ICP1-mediated PLE transduction experiments

PLE	Strain	Integration Information
PLE 1	KS518	In VCR in between VCA0336 and VCA0337
	KS519	In VCR in between VCA0329 and VCA0330
	KS520	In VCR in between VCA0329 and VCA0330
	KS441	In VCR in between VCA0362 and VCA0363
	KS443	In VCR in between VCA0354 and VCA0353
PLE 2	KS543	Interrupting VCA0581
	KS544	Interrupting VCA0581
	KS545	Interrupting VCA0581
	KS546	Interrupting VCA0581
PLE 3	KS551	In VCR in between VCA0301 and VCA0302
	KS552	In VCR in between VCA0435 and VCA0436
	KS553	In VCR in between VCA0415 and VCA0416
	KS554	In VCR in between VCA0463 and VCA0466
PLE 4	KS845	In VCR between VCA0353 and VCA0354
	KS846	In VCR between VCA0415 and VCA0416
	KS847	In VCR between VCA0407 and VCA0408
PLE 5	KS704	In VCR in between VCA0407 and VCA0408
	KS705	In VCR in between VCA0443 and VCA0463
	KS795	in VCR in between VCA0407 and VCA0441
	KS797	In VCR in between VCA0407 and VCA0408
