# Supplementary material for: A highly specific phage defense system is a conserved feature of the Vibrio cholerae mobilome
Source: PLoS Genet. 2017 Jun 8;13(6):e1006838. doi: 10.1371/journal.pgen.1006838 (PMC5481146; doi:10.1371/journal.pgen.1006838)
Supplement: S4 Table — (PDF) [file pgen.1006838.s008.pdf]

S4 Table. Bacterial strains, plasmids and phages used in this study

Strain/phage name	Description[a]	Source
***V. cholerae* strains**		
E7946	*V. cholerae* O1, El Tor biotype; SmR, is used as the PLE$^-$ control	[1]
KS393	*V. cholerae* clinical Isolate from the ICDDR, B, harbors PLE 1	[2]
KS393 S8*	*V. cholerae* clinical Isolate from the ICDDR, B, harbors PLE 1. Target of ICP1_2011 CRISPR spacer 8 target is mutated	[2]
KS229	*V. cholerae* clinical Isolate from the ICDDR, B, harbors PLE 2	[2]
KS511	*V. cholerae* clinical Isolate from the ICDDR, B, harbors PLE 3	[3]
MJ-1236	*V. cholerae* clinical Isolate from Matlab, Bangladesh, harbors PLE 4	[4]
O395	*V. cholerae* O1, classical biotype, SmR, isolated in India 1965, harbors PLE 5	[5]
KS393 S8*, PLE 1-Kan^R	KS393 S8*, Kanamycin resistance cassette inserted downstream of ORF 23 in PLE 1	This study
KS393 S8* Δ *int*, PLE 1-Kan^R	KS393 S8* PLE 1- Kan^R containing an in-frame deletion of PLE 1 integrase (constructed by flp-FRT recombination)	This study
KS229 PLE 2-Kan^R	KS229, Kanamycin resistance cassette inserted downstream of ORF 27 in PLE 2	This study
KS511 PLE 3-Kan^R	KS511, Kanamycin resistance cassette inserted downstream of ORF 27 in PLE 3	This study
MJ-1236 PLE 4-Kan^R	MJ-1236, Kanamycin resistance cassette inserted downstream of ORF 29 in PLE 4	This study
O395 PLE 5-Kan^R	O395, Kanamycin resistance cassette inserted downstream of ORF 29 in PLE 5	This study
KS683	E7946, PLE 5-Kan, generated via natural transformation of E7946 with gDNA from O395, PLE 5-Kan^R	This study
KS790	E7946, PLE 5-Kan. Target of ICP1_2011 CRISPR spacer 1 target is mutated	This study
KS843	E7946, PLE 4-Kan, generated via natural transformation of E7946 with gDNA from MJ-1236, PLE 4-Kan^R	This study
PLE 1	E7946 containing PLE 1 integrated in VCR in between VCA0329 and VCA0330	This study
PLE 2	E7946 containing PLE 2 interrupting VCA0581	This study
PLE 3	E7946 containing PLE 3 integrated in VCR in between VCA0415 and VCA0416	This study
PLE 4	E7946 containing PLE 4 integrated in VCR in	This study

	between VCA0353 and VCA0354	
PLE 5	E7946 containing PLE 5 integrated in VCR in between VCA0407 and VCA0408	This study
PLE 1-Kan^{R}	E7946 PLE 1, Kanamycin resistance cassette inserted downstream of ORF 23	This study
PLE 2-Kan^{R}	E7946 PLE 2, Kanamycin resistance cassette inserted downstream of ORF 27	This study
PLE 3-Kan^{R}	E7946 PLE 3, Kanamycin resistance cassette inserted downstream of ORF 27	This study
PLE 4-Kan^{R}	E7946 PLE 4, Kanamycin resistance cassette inserted downstream of ORF 29	This study
PLE 5-Kan^{R}	E7946 PLE 5, Kanamycin resistance cassette inserted downstream of ORF 29	This study
E7946 Δ *lacZ*::Spec^{R}	E7946 containing a deletion/insertion mutation where the lacZ gene was replaced with a SpecR cassette	This study
PLE 1 ΔVC1807::Kan^{R}	E7946 PLE 1 containing a deletion/insertion mutation where VC1807 was replaced with a KanR cassette	This study
PLE 2 ΔVC1807::Kan^{R}	E7946 PLE 2 containing a deletion/insertion mutation where VC1807 was replaced with a KanR cassette	This study
PLE 3 ΔVC1807::Kan^{R}	E7946 PLE 3 containing a deletion/insertion mutation where VC1807 was replaced with a KanR cassette	This study
PLE 4 ΔVC1807::Kan^{R}	E7946 PLE 4 containing a deletion/insertion mutation where VC1807 was replaced with a KanR cassette	This study
PLE 5 ΔVC1807::Kan^{R}	E7946 PLE 5 containing a deletion/insertion mutation where VC1807 was replaced with a KanR cassette	This study
PLE 1 Δ *int*, PLE 1-Kan^{R}	E7946 PLE 1- Kan^{R} containing an in-frame deletion of PLE 1 integrase (constructed by flp-FRT recombination)	This study
E7946 Δ *wbeL* Δ *lacZ*::Spec^{R}	E7946 containing a clean deletion of *wbeL* and a deletion/insertion mutation where the *lacZ* gene was replaced with a SpecR marker	This study
KS227	*V. cholerae* clinical isolate from the ICDDR, B	[3]
A50	*V. cholerae* O1, classical biotype, SmS, isolated in Bangladesh 1963	[6]
A57	*V. cholerae* O1, classical biotype, SmS, isolated in India 1980	[6]
A68	*V. cholerae* O1, classical biotype, SmS, isolated in Egypt 1949	[6]
A111	*V. cholerae* O1, classical biotype, SmS, isolated in 1990	[6]

B33	*V. cholerae* clinical Isolate from Beira, Mozambique	[4]
KS510	*V. cholerae* clinical isolate from the ICDDR, B	[3]
KS409	*V. cholerae* clinical isolate from the ICDDR, B	[3]
KS396	*V. cholerae* clinical isolate from the ICDDR, B	[3]
KS398	*V. cholerae* clinical isolate from the ICDDR, B	[3]
KS400	*V. cholerae* clinical isolate from the ICDDR, B	[3]
KS401	*V. cholerae* clinical isolate from the ICDDR, B	[3]
KS344	*V. cholerae* clinical isolate from the ICDDR, B	[3]
KS255	*V. cholerae* clinical isolate from the ICDDR, B	[3]
KS348	*V. cholerae* clinical isolate from the ICDDR, B	[3]
KS517	*V. cholerae* clinical isolate from the ICDDR, B	[3]
KS516	*V. cholerae* clinical isolate from the ICDDR, B	[3]
KS515	*V. cholerae* clinical isolate from the ICDDR, B	[3]
KS39	*V. cholerae* clinical isolate from the ICDDR, B	[3]
KS518	E7946 PLE 1-KanR. E7946 added to supernatants of KS393 S8* PLE 1-Kan^R infected with ICP1_2011_A ΔS9	This study
KS519	E7946 PLE 1-KanR. E7946 added to supernatants of KS393 S8* PLE 1-Kan^R infected with ICP1_2011_A ΔS9	This study
KS520	E7946 PLE 1-KanR. E7946 added to supernatants of KS393 S8* PLE 1-Kan^R infected with ICP1_2011_A ΔS9	This study
KS441	KS227 PLE 1-KanR. KS227 added to supernatants of KS393 S8* PLE 1-Kan^R infected with ICP1_2011_A ΔS9	This study
KS443	KS227 PLE 1-KanR. KS227 added to supernatants of KS393 S8* PLE 1-Kan^R infected with ICP1_2011_A ΔS9	This study
KS543	E7946 PLE 2-KanR. E7946 added to supernatants of KS229 PLE 2-Kan^R infected with ICP1_2006_E	This study
KS544	E7946 PLE 2-KanR. E7946 added to supernatants of KS229 PLE 2-Kan^R infected with ICP1_2006_E	This study
KS545	E7946 PLE 2-KanR. E7946 added to supernatants of KS229 PLE 2-Kan^R infected with ICP1_2006_E	This study
KS546	E7946 PLE 2-KanR. E7946 added to supernatants of KS229 PLE 2-Kan^R infected with ICP1_2006_E	This study
KS551	E7946 PLE 3-KanR. E7946 added to supernatants of KS511 PLE 3-Kan^R infected with ICP1_20011_A	This study
KS552	E7946 PLE 3-KanR. E7946 added to supernatants of KS511 PLE 3-Kan^R infected with ICP1_20011_A	This study
KS553	E7946 PLE 3-KanR. E7946 added to supernatants of KS511 PLE 3-Kan^R infected with	This study

	ICP1_20011_A	
KS554	E7946 PLE 3-KanR. E7946 added to supernatants of KS511 PLE 3-Kan^R infected with ICP1_20011_A	This study
KS845	E7946 PLE 4-KanR. E7946 added to supernatants of KS843 infected with ICP1_20011_A	This study
KS846	E7946 PLE 4-KanR. E7946 added to supernatants of KS843 infected with ICP1_20011_A	This study
KS847	E7946 PLE 4-KanR. E7946 added to supernatants of KS843 infected with ICP1_20011_A	This study
KS704	E7946 PLE 5-KanR. E7946 added to supernatants of KS683 infected with ICP1_20011_A ΔS9	This study
KS705	E7946 PLE 5-KanR. E7946 added to supernatants of KS683 infected with ICP1_20011_A ΔS9	This study
KS795	E7946 PLE 5-KanR. E7946 added to supernatants of KS790 infected with ICP1_20011_A ΔS9	This study
KS797	E7946 PLE 5-KanR. E7946 added to supernatants of KS790 infected with ICP1_20011_A ΔS9	
**Phage**		
ICP1	O1-specific phage of *V. cholerae* O1, unclassified member of the *Myoviridae* family	[7]
ICP1_2004_A	CRISPR-Cas positive ICP1 isolate	[7]
ICP1_2005_A	CRISPR-Cas positive ICP1 isolate	[7]
ICP1_2006_E	CRISPR-Cas positive ICP1 isolate	[2]
ICP1_2011_A	CRISPR-Cas positive ICP1 isolate	[2]
ICP1_2011_A ΔS9	Spontaneous deletion of spacer 9 in phage CRISPR array	[2]
ICP1_2004_A Δ*cas2-3*	ΔCRISPR derivative constructed by deleting *cas2-3*	This study
ICP1_2005_A ΔCRISPR	ΔCRISPR derivative constructed by deleting CRISPR arrays (CR1 and CR2) such that no anti-PLE spacers remain	This study
ICP1_2006_E ΔCRISPR	ΔCRISPR derivative constructed by deleting CRISPR arrays (CR1 and CR2) such that no anti-PLE spacers remain	This study
ICP1_2011_A Δ*cas2-3*	ΔCRISPR derivative constructed by deleting *cas2-3*	[8]
ICP2	*V. cholerae* phage, member of the *Podoviridae* family	[7]
ICP3	*V. cholerae* phage, member of the *Podoviridae* family, T7-like phage	[7]

REFERENCES

1. Levine MM, Black RE, Clements ML, Cisneros L, Saah A, Nalin DR, et al. The pathogenicity of nonenterotoxigenic *Vibrio cholerae* serogroup O1 biotype El Tor isolated from sewage water in Brazil. J Infect Dis. 1982;145: 296–299.

2. Seed KD, Lazinski DW, Calderwood SB, Camilli A. A bacteriophage encodes its own CRISPR/Cas adaptive response to evade host innate immunity. Nature. 2013;494: 489–491. doi:10.1038/nature11927

3. Dalia AB, Seed KD, Calderwood SB, Camilli A. A globally distributed mobile genetic element inhibits natural transformation of *Vibrio cholerae*. Proc Natl Acad Sci USA. 2015;112: 10485–10490. doi:10.1073/pnas.1509097112

4. Grim CJ, Hasan NA, Taviani E, Haley B, Chun J, Brettin TS, et al. Genome sequence of hybrid *Vibrio cholerae* O1 MJ-1236, B-33, and CIRS101 and comparative genomics with *V. cholerae*. J Bacteriol. 2010;192: 3524–3533. doi:10.1128/JB.00040-10

5. Taylor RK, Miller VL, Furlong DB, Mekalanos JJ. Use of phoA gene fusions to identify a pilus colonization factor coordinately regulated with cholera toxin. Proc Natl Acad Sci USA. 1987;84: 2833–2837.

6. Mutreja A, Kim DW, Thomson NR, Connor TR, Lee JH, Kariuki S, et al. Evidence for several waves of global transmission in the seventh cholera pandemic. Nature. 2011;477: 462–465. doi:10.1038/nature10392

7. Seed KD, Bodi KL, Kropinski AM, Ackermann H-W, Calderwood SB, Qadri F, et al. Evidence of a dominant lineage of *Vibrio cholerae*-specific lytic bacteriophages shed by cholera patients over a 10-Year Period in Dhaka, Bangladesh. mBio. 2010;2: e00334–10–e00334–10. doi:10.1128/mBio.00334-10

8. Box AM, McGuffie MJ, O'Hara BJ, Seed KD. Functional analysis of bacteriophage immunity through a Type I-E CRISPR-Cas System in *Vibrio cholerae* and its application in bacteriophage genome engineering. J Bacteriol. 2016;198: 578–590. doi:10.1128/JB.00747-15
