# Supplementary material for: A highly specific phage defense system is a conserved feature of the Vibrio cholerae mobilome
Source: PLoS Genet. 2017 Jun 8;13(6):e1006838. doi: 10.1371/journal.pgen.1006838 (PMC5481146; doi:10.1371/journal.pgen.1006838)
Supplement: S1 Dataset — (RTF) [file pgen.1006838.s009.rtf]

>PLE1_ORF_1_IntegraseMPKLYIFRRVSSKRQTNKTGLKIQVRPQTVERLLSEHGLSEVVDLGAEIASASKGKHIEEGTILKRFLTKCERGEIEQGSILAVYSLDRLSRLEKLGEAKSLIYLPITNNGVSIFGEIDNFLYKPHDVLSEILSSLVFERAAEESRAKKNRNIDALAANLAQYQRDGQYTTKLGKSPSWINHKTGKLNEEAVLWREVINCYTSGQYKNLSALHTAMQLPIPYNTLSKVINNAHKTLIGTKVIKGVELVEFYEPLLTEEEYNRLLTHVRQMGTGKGKAVATTKTENIWLLKGLAVCPECGSLCGSFKNGASVKDKVRYNYRCNAAINKHCNSPTYNLNPIEAIVTLLSADYLHEVSQRETNTELNAKIEATQVEVERLETQLERAGVLINEYLSEELVKAIPKYREQLENAQNSLQTLLALASNHTPIDERLLSAEIILDKANPERMKLVKLLQGCINRVIVTRKSVKKGDTRFSPKIYTGCDYSLLCVRVEFVDGNIRVFDSVLRDNLRLNLSVSNGTRSIGLDNFHRFSRRLREYDLIKYCVINNRKLKIT*>PLE1_ORF_2_MKITRLQREFIGEQFHTPKGGTLTVTGITDQTSGRNAVFTVECSICSVDEVLFPDGFTSTKSNLVCNERVPCPCSGRYKYSPNQYHILVQRNCTQKGYTLLEFGGEVGEWLGTTKTPITLLNPKTGRTWTTTVYGFLNT*>PLE1_ORF_3MTAQTFNFEYDEYNRLLLPLQYHDITCAFVDALGFRVEDLVGQSNQVRMSLISLIPPQTIVEIKQLVELHHDYFKRWLTEENKCIFNDILHNFIKAYTTTTAKASTYGSGISWQAINAETFKLMIELIDSK*>PLE1_ORF_4_MSNVYPVYKSNDNIKASLDNHLDTLLEAAKGSNKALGRINKAYDGIMLQLIKACKGEIELTKGQSDAIKIVLNEQKHLSKVVADLEELLKKVQAVEAGQQPTTTPNTPPKPFDPRQFKSTKF*>PLE1_ORF_5_MSSKILSKIQNDIIGLGMSLMAETRTNDVTKLVVCLSGLNIPRATIANIVKAETGTTLSVNRITKIRSAYSSIVKTLSEETDHLYQFHDIA*>PLE1_ORF_7_MQHNTPYLPHQSLQLQSRLIKLYQNVGLFVPKLTFCFSVDNAETDTHYLNVWFSSNVDGELIIGECNTYEKAALIAQQWLDDISDDDLLHIVQSVLSRGYTDGFSECVEGVRQADKLTSNELLVLECLRPQLDFEEGSESGQYPWSNTDISDKTGIHRSNLHRVLKSLVNKGLIVEETVKRDVWASLKNCGHVQKDVLVYYPVATREEDLEVMENWHEWRAIRQKEAMQKWRAMLSS*>PLE1_ORF_8_MDERFNAALHESAHTVIAQVLGFNTATPIIYENSSTNPDEKHWLGKAFIDTTNGNVEDIALVGLAGEAIQYYIEGVDVGDCPFIWECNLEDISLSDQELVKDLYNDVELWEKLYTLFEQHHDSILDLANSI*>PLE1_ORF_9_MKYEIPPSLNLKELPLTTQYQLNRMLNGEIRPSAIRRNKANYKLKGDKDKVFENGLAVRLFNLIREYNNVESVESEEV*>PLE1_ORF_10_MKIQLKHPYDKLAGELADLTGLSPTQLTIQLMQELQTKISKTSAQEEAKENNNNVKVLPN*>PLE1_ORF_11_MSKFYQINTTLLESNEAVNKQTGEVVPLSPETKLVYAYMLNQYRMYRKYGNRRYTESWDKIFTVCCDVAAQKQKRLAKELTTLGLIEVIGNKNAYKVVHSVESIIETWEFTNSKLNTFKDPTQTTKRKESKKQRLNKWEEDKRSKGEWKDFKEPHLEEMQDCNGVESYFTEQPPVEAYEQDYRPIAGDAVNFGVLADIDTSDAPTSDQWDYHPPEEKPEEVEPQEALEVIEQQVVEQDEPKKDEPQEVVKPITPVVCGDKSKLLKCFRPNTDISEIDISGKGIGFFAKPLHLKECDKDTARQYLKVIKEYYWEQYNPDDVTIANMVSWLTCSVCSNLTNELGYCLDPNCSSNNLPF*>PLE1_ORF_12_MNNAETRLLQIIETDEAKWGYSEGCEVGDFKSLRTDKGKKEYFDELTHNRPIYSLIED*>PLE1_ORF_12.1MNIQDVTAAIDLLTIAYVFIKSHPYYTEFMQYLLLNQQ*>PLE1_ORF_13_MNPDYVKLKLKQGQIDSINKKIAKLAKKNTPNAVVKKLTTQCLQGLLTYDHEVKVLMEYVVANADCFVDEQVKCAKKYLAAKTAPYPILFLMDFYNAGSSDGWNYKNVLLGI*>PLE1_ORF_14MYQVEFKTKEGARYTVVFTQYVCAMPNPDTLHVTSDRFDFKITRIKNDRSITQDVNFNLKIDGKVIPATQYVVSGQNSKRTSKQTDVILSNYYNWLDLI*>PLE1_ORF_15_MPTQKTLDRAAKINCTIKQLQGVYAQYKNIKYRCGKGRYNNAKFLLGNFDTFIEWIASQGGYRQSPHGHAYHVSRIRDQGDYAYNNIRWCSHQENLKETSNFYLVTDLKSSRQELFQGNLADKLKELFNVEATSGAYTASKTGNLYLGRFKIDRLEI*>PLE1_ORF_15.1_MITIELLNSKLAMLSQTERQALRLMLTTNLTNSEINKELGVSHNYVYWLKNESPHAETVQMFLNLNNKFKETKE*>PLE1_ORF_16MITINLKLGNSESITRTYSQISIEETRIVLDTPMESVNETVLSFANGVLSSITVTEPKQGVSTMPQATTRSAVYATISNKGLVLNVTTYATLSTLLSGISANAEIVKSLKQKPTQQQVVKSSLGKETQEQTQQRIQRALAAKQSKQANTGVNKQQIYDELNKARAKDAQPLMDIVKAKQTELIKCASAKAQTAEWGELVKQLSGKKFTPEAFDAFREVLTISDITKCERMVRELANRLNIVNGVIHLIK*>PLE1_ORF_17_MTTITTTTNNTFDLANVIAEYKAGFEQYKADNKQYNADAYRRKIESINSDAALTNGAFNQFAYGSQMFEGKTLQEIAESLKTMQVKDSSREDENGLIFPHVTLQLVSPTTPAQYYGLIAEAVKLGFEVCPDWRLHVGTGRNFPACRLVRQAEWYKPHNEKLMAERIAEAEKQEAERLKAEYFNEHRVQAYVEQAQRKFMATQAQQAAISLSAAISRELYASSGLSDDDLAVVAQSDVWAFNTLAPQLQEKDPNVISAALTGAGFVKGKHKLSDGKQATLWVKDGADVTALTLESKYIQ*>PLE1_ORf_18_MKMTAEQFNERLAHIVKCWVEFGYEVETWEEFLAAELKGMELQAELTAEVEQRLQYALNSLKTLKLIGGE*>PLE1_ORF_18.1_MFDPRFEMNPEIEYLLEEIAAHNQRLKNCGINPDHDTHMQEMQERVQHLLRLHLSAVNKFMGYTKEDNK*>PLE1_ORF_19_MYTNTIIKTEIDEKVIKAFKLDALTRSKLFFKLTTKLAVPFAGVIDGAFSADRSLVCASVASLLSQHLDQETFEETQLILFGSIVEDGEALATPEAINKWFEYNDVNPIDLFVWLVDENLVTLFKGSKQLQSLKPKFDEFYKKFEDFIPQTVISDDKAEE*>PLE1_ORF_20_MIKLKSDKLEIVKGELSIGDVRWLFEDKDALEHVYSNLNREDKQRVDESPLSLPTLADAYLKHCLQTVAESPRLPTRSEWNLANKRAAALKLAENNPTRIQMLNEIEEAERKQNNISNPNAHSGIKRNFLKWLEDNARTLSPFDQWINSVAAYANMSPAQCLQLPYKAFITLSNSYTIEQEQKHVMQQEREKQDALRHN*>PLE1_ORF_21_MNIEKYIESKRVVLDKMHALVNIAPKINKDQHGNITYSEDNKYKTLSMQLADELELYHLICNPLYAETVNASYTQQIDDIATALYINRDNEEFTDLIVEFCMGNPTYTKLFS*>PLE1_ORF_22_MAENLVRYGASLAFDGRQALRQLRKYKETFNKVQHTILKNTQKLNNEMVQGEQRVQRAKENSIEKEKRRQESFNQWRKRQTRSANFAELKAHQQVELMKILASNKSAQEIKSTYTDTLLAFKLAKKREVAAEKQANRLRLESAKRTETRIKAIKGRANNLLGRAGGVAGSAISGLLGLGAATLGTVAVGAKDASDTRAEAVNAGMKVEDYQRFLFGARNATNLSRDEIMSSLQDVNDRRGEIMNLEIKDGEIKGVGELTDLLNFLVKNGQLALDEKAIKDFVLNSGNSAEFLEKVFKLLQTSGADTNTQTFLMESLASNSFRLMAQIDQNSTMYARSLEEMKTLRLGIDAANQTQVRDVGQTMSAMWNKLTYLPLEAFEAFAMAIKPETAASMNSVVNSIIKLARAMGDDLANVLERIVPWLEKLLASANENSLIDNFRSVGNILFDEIVKPALVKVGEVLLGVVATAIPEGMRTDQLQQMYEKYMLTEEERQALQLKKQQELDLNWQRIQPKQGGFNYLTPPPQAKTDTSSYVYPSAQQPVVVENNSTVKLEVDGKVLAQTVTKDQQFNEAMRKAAHQYGRGGY*>PLE1_ORF_23_MMYFQTLNGELFCLDATNAIDISRRAMVTESPILTQKDRTEGYSVGNRRVEVQGLITYTKSVRQQFVGNPTPDKAIKLINGVMDNHEYFTVFWDNLGKYKLAGDPIKNCVVESFRYVIDEFLDTITVSLILKEIDIRDADGKGSLKETPSPEASSQISDEQTVSSGGVWQTKEQTRSEWLRLYEDLVKASNPNTAKQAITGMVAAD*>PLE2_ORF_1MQNVNSKQTLKAIYVTVSDVELLRFVNNCNRHLTVRQAVKLRLRIARMHYRNSQLN*>PLE2_ORF_2_INTEGRASE_MQPSIYVYRRVSTERQAKADKHGLDIQVRESTLIDLQKQFPNYPVVELANDAGLSAFKAEHIKVKTALKDFIDKCVNGEIVRGSILVVYSLDRLSRLKLGDAYQKVFIPIVENGVQIYSETEARFYSNHDVDYILATVLFSRAHNESKTKSQRNKDAILNGVKRWQQEKKFTPNLTTSPFWIDNKTGEFNHLADAALYMVESLINGVGYKTIADELERCYPTPPQRKTSKAPSFWTLETIFKTRNNRSLIGEKTIKVQKDLEISLEKPEKVKPKKNGEKVVKDKKEKAEDTYTLDGYYPSLISMDTWHKLQKTKANKRRTYSDNIYLLTDLRSLAVCGECGYSLAGTANKGGSDERAKYFCTGASSKRSVHTLWKADVETMDRVVLLLLNAGIEEGVKLITSPEYKSMIEDLKNTILSEQHTLSELKTRFERTQSHTMLDLMLTAEDKIKQAEKRLEELTASPTNATGDNITDYEIEFYRLYRETNFKDLTDITRKDIKLEIAKLLQRVEISPCGCGWRISLKMNTGVCVEYVTTQAITTDTINMGNKYIRQELAYNGLYHYMIRVKNSRTMENHRIIEKWLRKTAQLDNIPSKPNFINDIIHEIEPVNPLRDSTKYDLTYADLELMFKR*>PLE2_ORF_3MTDTLEMKLTETQAERVIAVLKSRVNIRELVEVAAIDINDLKGIRDRLSSVITEIENAGLIADCEKAIDQLRKVGVPVPKELYSKLRSLQGGNVGDKPSTKKGEGKRNIKTGVYLVEGYEPLNASSVGAPSKQLKDLMKATGLSRQELNEKYFTPDSEE*>PLE2_ORF_4MKITKGQKEFIGTQFPTPKGGTLTVTGITDQTSGHNAVFTLECSICGVDEVLFPDGFTSTKSNLVCNARVPCSCSGRYKYSPNQYHILVQRNCAQKGYTLLEFGAESGEWFGAAKTPITLLNPKTGRTWTTTVYGFLNT*>PLE2_ORF_5MNLLDEFKPVLKIKRQCIGNCSMYRVLLAVGYTQQELQADYGIYQPLISLIPIGAESEINSFLDGKLEIFNALLQQVGFDAMRYTLQQWFAGLENDGRYMDYSLMDSSWENVKKSVIPLVLNPEVKFEYV*>PLE2_ORF_6MLKPLPIQSSTQGRVTKRKEINVLCAEVTQEKIAAVDKIKGKIPKMIKQLQDIIDHPDTTDSNIMKAIAQLTAMLERWESDLTKIKNDEEYLDTTTEQQQQQQPTTTRKNSSTVDIMALLEKDGK*>PLE2_ORF_7MTDLKSKKLIQIQNEIFSLCKILMKQHYRSNKKTAAIVAMLGLNLTGSQVVEMMQEIEGEKVSLSSVHKARERYRPIVKMLQEETNRLYSLHGFI*>PLE2_ORF_8MTKRTRRLFSAEFKLEAAQLVLVQNYSVTEAAQAMNVGKSTMDKWVRQLKQERQGKAPKASPMTPEQIEIRELKKKLARLEEHNEILKKSHGSLDVGLTEQPTVSLCSLYVVFV*>PLE2_ORF_9MQHNTPYLPHQSLQLQSRLIKLYQNVGLFVPKLTFCFSVDNAETDTHYLNVWFSSNVDGELIIGECNTYEKAALIAQQWLDDISDDDLLHIVQSVLSRGYTDGFSECVEGVRQADKLTSNELLVLECLRPQLDFEEGSESGQYPWSNTDISDKTGIHRSNLHRVLKSLVNKGLIVEETVKRDVWASLKNCGHVQKDVLVYYPVATREEDLEVMENWHEWRAIRQKEAMQKWRAMLSS*>PLE2_ORF_10MNLVKKHQLTRVQQTFVKNHLNDFIDNIKYQVHESMGSCHTVALRTLPVVN*>PLE2_ORF_11MRECWQSGKGYASGNMFLAVCEWLEWNEQSESEDSFETFPYFGGSEFAAEMVDILLKEELPEETLTVYRGGDLDGWSWSTNKAVALQFANPPLEAKRPLYQLEVNTKDIITKLTDRNESEVVINPAILETLSPIELNNNL*>PLE2_ORF_12MTPTEIQNHINIFLVAKYYEILKEHPEKYARKRYYYKKSCQADHYIALGVAHALIESEV*>PLE2_ORF_13MKIQLDNETLDLVMPYCKSNSVKPHQLIKTLLMQFMQQPVTHDEEKKSDNTN*>PLE2_ORF_14MTTPTNTEVKTYSTKDSNGKTQYQMHTEVSLADCGTYGLNNSDKAFLLAILADARWDGNKWESKLSIPMIQKRSGMGEKAQRASRKRLIELGVIKAYQQFDNSLIFHFRSLDLNEVRVQEFAEALAKKTDGKILHIKVSENNLLLKRYKKSWGETYPKRYESFKNAVMAKTRNGEIFNSEHHKAWRKTYTLNNPDDVMDLDLIYLTDGDMVVIGENVKVINNGIEKPIVLKQESAPKPFSLKAGETQNDYDHAFDGIEPTFEDVPTSEEEPEEVQPQEALEVIEQQVVEQDEPKEVVEVVTPQVVEHVTPVVCGDKSKLLKCFRPNTDISEIDISGRGIGFFAKPLHLKECDKDTARQYLKVIKEYYWEQYNPDDVTIANMVSWLTCSVCSNLTNELGYCLDPNCSSNNLPF*>PLE2_ORF_15MNIQDVTAVIDFITVSYIFVKSHPYYIQLCEFFLHQQ*>PLE2_ORF_16MTPDYVKLKLKQGQIDSMCKKIDKLAKKNTPNAVVKKLTTQCLQGLLTYDHEVKVLMEYVVANADCFVAEQVKCAKKYLAAKTAPYPIMFLMDFYNAGLEDGWNYKNVLLGI*>PLE2_ORF_17MPTQKTLDRAAKINCTIKQLQGVYAQYKNIKYRCGKGRYNNAKFLLGNFDTFIEWIASQGGYRQSPHGHAYHVSRIRDQGDYAYNNIRWCSHQENLKETSNFYLVTDLKSSRQELFQGNLADKLKELFNVEATSGAYTASKTGNLYLGRFKIDRLEI*>PLE2_ORF_18MITIELLNSKLAMLSQTERQALRLMLTTNLTNSEINKELGVSHNYVYWLKNESPHAETVQMFLNLNNKFKETKE*>PLE2_ORF_19MITINLKLGNSESITRTYSQISIEETRIVLDTPMESVNETVLSFANGVLSSITVTEPKQGVSTMPQATTRSAVYATISNKGLVLNVTTYATLSTLLSGISANAEIVKSLKQKPTQQQVVKSSLGKETQEQTQQRIQRALAAKQSKQANTGVNKQQIYDELNKARAKDAQPLMDIVKAKQTELIKCASAKAQTAEWGELVKQLSGKKFTPEAFDVFKEVLTISDITKCERMVRELANRLNIVNGVIHLIK*>PLE2_ORF_20MTTTTNINTTFEISAVIAEYKAGFEQYKADNKQYNADAYRRKIESINSDAALTNGAYRDFAYGSQLFEGDKTLTEIAEMLKTRTAQTEREDEQGFIYPHVVIQFVSPMTATQYYGLIAECVKLDFEVCPDWRLHVGTGRQFPACRLVRKAEWYKPHNEKLMAERIAEAEKQEAERLKAEYFNEHRVAAYVEQAQRKFMAAQAQQAAISLSAAISRELYATSGLGDDDLAVVAQSDVWAFNTLAPQLQEKDPNVISAALTGAGFVKGKHKLSDGKQATLWVKADADVTATDSKFI*>PLE2_ORF_21MKMTAEQFNERLAHIVKCWVEFGYEVETWEEFLAAELKGMELQAELTAEVEQRLQYALNSLKTLKLIGGE*>PLE2_ORF_22MFDPRFEMNPEIEYLLEEIAAHNQRLKNCGINPDHDTHMQEMQERVQHLLRLHLSAVNKFMGYTKEDNK*>PLE2_ORF_23MYTNTIIKTEIDEKVIKAFKLDALTRSKLFFKLTTKLAVPFAGVIDGAFSADRSLVCASVASLLSQHLDQETFSSTQLILFGSIVEDGEALATPEAINKWFEYNDVNPIDLFVWLVDENLVTLFKGSKQLQSLKPKFDEFYKKFEDFIPTTVISDDKAEE*>PLE2_ORF_24MIKLKSDKLEIVKGELSIGAVRWLFEDVDALEHVYSNLNREDKQRVDESPLSLPTLADAYLKHCLQTVAESPRLPTRSEWNLANKRAAALKLAEDNPTRIQMLQEIDEAERKQNNISNPNAHSGLKRNFLKWLEDNARTLSPFDQWINGVAAYANMSPAQCLQLPYKAFITLSNSYTIEQEQKHVMQQEREKQDALRHN*>PLE2_ORF_25MNIEKYIESKRVVLDKMHALVNIAPKINKDQHGNITYSKDNKYKTLSMQLTDELELYHLICNPLYAETVDASYTQQIDDIATALYINRANEEFTDLIVEFCMGNPTYTKLFS*>PLE2_ORF_26MAENLVRYGASLAFDGRQALRQLRKYKETFNKVQHTILKNTQKLNNEMVQGEQRVQKVKENTIEKEKRRQESFNQWRKKQMRSANFAELKAHQQVELMKIFASNKSAQEIKSTYTDTLLAFKLAKKREVAAEKQANRLRLESAKRTESRIKAIKARANNLLGRAGGVAGSAVSGLLGLGAAALGTVAVGAKDASDTRAEAVNAGMKVEDYQRFLFGARNATNLSRDEIMSSLQDVNDRRGEIMNLEIKDGEIKGVGELTDLLNFLVKNGQLALDEKTIKDFVLNSGNSAEFLEKVFKLLQTSGADTNTQTFLMESLASNSFRLMAQIDENSTMYQRSLEEMKNLRLGIDAANQTQVRDVGQTMSAMWNKLTYLPLEAFEAFAMAIKPETAASMNSIVNSIIKLARAMGDDLANVLERIVPWLEKLLASANENSLIDNFRSVGNILFDEIVKPALVKVGEVLLGVVATAIPEGMRTDQLQAMYEKYMLTDEERQALQLKKQKEMDLNWQRIQPKQGGFNYLTPPPQAKTDTSSYVYPSAQPAVVVENNSTVKLEVDGKVLAQTVTKDQQFNEAMRKAAWSVGR*>PLE2_ORF_27MMYFQTLSGELFCLDATNAIDISRRAMVTESPILTQKDRTDGYSVGTRRVEVQGLITYTKSVRQQFVGNPTPDKAIKLINAVMDNHEYFTVFWDNLGEYKLAGDPIKNCVVESFRYVIDEFLDTITVSLILKEIDIRDADGKGSLKETPSPEASSQISDEQAVSSGGVSQTKEQTRSEWLRLYEDLVKASNPVTAKQAITGMVAAD*>PLE3_ORF_1MRKQSQSLKPVVVTAQPCEIIRFANHSKHLSIKQCVRLRLLAARLEYRQSVI*>PLE3_ORF_2MFKVLIHERSKDVKILPDGGFDLTVLLFGVFVPLWRGQYKRALSYLAIVICTLFVAWVFIPFFSNKHYIKDLLEKDGYLLAEDYTGVKPVIPDTSPLMIMMKDFISWFMFAHVLLGVLGLFIFGDLLLAVIIIALFGLGLALRYHRALAKHQAITKYNEVSNAA*>PLE3_ORF_3MLLDQDYIDSNFRKYHINTIKKWCSGECRGLTEELALNSLDVLKNVEDFTLTEIAEATGVTLETVAKLNAKLVLS*>PLE3_ORF_4_IntegraseMPKLYIFRRVSSKRQTNKTGLKIQVRPQTVELLLAEHGLSEVVDLGAEVASASKGKHIEEGTILKRFITQCERGEIEQGSILAVYSLDRLSRLEKLGEAKSLIYLPITNNGVSIFGEIDNFLYKPHDVLSEILSSLVFERAAEESRAKKNRNIDALAANLAQYQRDGQYTTKLGKSPSWIDHRTGKLNEEAVLWREVINCYTSGQYKNLSALHTAMQLAIPYNTLSKVINNAHKTLIGTKVIKGVELAEFYEPLLTQEEYNRLLIYVRQMGTGKSKAVATTKTENIWLLKGLGECPQCGSLCGSFKNGASVKDKVRYNYRCNAAINKHCDSPTYNLNPVEAIVTLLSADYLHEVSQRETNTALNAKIEAMQVEVERLETQLERAGVLINEYLSEELVKAIPKYREQLETAQNELQTLLALASNHTPIDERLLSAEVILDKANPERMKLVKLLQGCINRVIVTRKSVKKGDTRFNPKIYTGCDYSLLCVRVEFIDGNIRVFDSVLRDNLRLNLSVSNGTRSIGLENFHRFSRRLREYDLIKYCVINNRKLKIT*>PLE3_ORF_5MKITRLQREFIGEQFHTPKGGTLTVTGVSPIKQGRGALFTVECSVCSADAELWPSGSIIASKGHLIKGVVPCGCTRSPRWTQDQFEILVKRKCEEKGYIFQGFVGEYKGAFTYLRLHNLQNDNTWETTTITSFLHIGTGCPLEARLKQKQQAV*>PLE3_ORF_6MNLLDEFKPVLKIKRQCIGNCSMYRVLLAVGYTKEELQSGYGIHQPLISLIPIGAESEINSFLDGKLEIFNALLQQVGFDAMRYTLQQWFAGLEKDGRYMDYSLMDSSWENVKKSVIPLVLNPEVKFEYV*>PLE3_ORF_7MLKPLPIQSSTQGRVTKRKEINVLCAEVTQEKIAAVDKIKGKIPKMIKQLQDIIDHPDTTDSNRMKAIAQLTAMLERWESDLTKIKNDEEYLDTTTEQQQPTTTRKNSSTVDIMALLEKDGK*>PLE3_ORF_8MTDLKSKKLIQIQNEIFALCKILMKQHYRSNKKTAAIVAMLGLNLTGSQVVEMMQEIEGEKVSLSSVHKARERYRPIVKMLQEETNRLYSLHGFI*>PLE3_ORF_9MTPTEIQNHINIFLVAKYYEILKEHPEKYARKRYYYKKSCQADHYIALGVAHALIESEV*>PLE3_ORF_10MKIQLDNETLDLVMPYCKSNSVKPHQLIKTLLMQFMQQPVTHDEEKKSDNTN*>PLE3_ORF_11MTTPTNTEVKTYSTKDSNGKTQYQMHTEVSLADCGTYGLNNSDKAFLLAILADARWDGNKWESKLSIPMIQKRSGMGEKAQRASRKRLIELGVIKAYQQFDNSLIFHFRSLDLNEVRVQEFAEALAKKTDGKILHIKVPENNLLLKRYKKSWGETYPKRYESFKNAVMAKTRNGEIFNSEHHKAWRKTYTLNNPDDVMDLDLIYLTDGDMVVIGENVKVINNGIEKPIILKQESSPKPFSLKAGETQNDYDHAFDGIEPTFEDVPTSEEEPKKDEPQEVVEVVTHQEVVKPITPVVCGDKSKLLKCFRPNIDISEIDISGKGIGFFAKPLHNKECDKDTARQYLKVIKAYYWDQYNPDNATIANMVSWLTCSVCSNLTNELGYCLDPNCSSNNLPF*>PLE3_ORF_12MSELTFKFENTTVTINTHEIEGQTLYKAQDLLKGYGMDTKKCNDTLRNWKNSKTVEFTVLKGRYGGTYLTKRQCLKLASYVSEDFEEAVYEAFEAAASGDGNKAVDIATSKVITPELLNKLNFWTPLLHKEITAWSARNRKGKFGYTMIYNHIVNKVVTNIYTKELKKSHSISSMKDYLIKQNHVEGLGAYIAMCELLVPLLQANSDYELLKSVFVETPKLYKAA*>PLE3_ORF_13MKYFQIDELTLNAMLRITTIESLTPEQRLELIKAHLLNIKTPSDDNEPWDEF*>PLE3_ORF_14MYQIEDGQAYIIVSRAEFDIYNALNDEGKKEYFDELTHNRPIYSLIED*>PLE3_ORF_15MNIQDVTAAIDLLTIAYVFIKSHPYYVEFMQYLLNQ*>PLE3_ORF_16MTPDYVKQKLKQGQIDSINKKIAKLAKKNTPNAVVKKLTTQCLQGLLTYDHEVKVLMEYVVANADCFVAEQVKCAKKYLAAKTAPYPILFLMDFYNAGLEDGWNYKNVLLGI*>PLE3_ORF_17MYQVEFKTKEGARYTVVFTQYVCAMPNPDTLHVTSDRFDFKITRIKNDRSITQDVNFNLKIDGKSVPATQYIVSGQNSKRTSKQTDVILSNYYNWVDLI*>PLE3_ORF_18MPTDNRKNLERAAKINCTLPQLRKLNVQFLNIKYRCGRLKGYETCQFKLGTFDDFINWIADQGGYRTSPHGHAYHVSRIRDQGDYAYNNIRWCSHQENLKETAHFWLVTDLLTSRQELFQGNLADKLKELFDVSGYGAYKAAESGRLYKNRFKIDKLSI*>PLE3_ORF_19MITIELLNNKLSELCPTARKCLRLMLTTNLTNSEINKALGLSHNYVYWLKNESPHAETVQMFLQLNNKFKETKE*>PLE3_ORF_20MITINLKLGNNESITRTYSQISIEETRIVLDTPMDTVNETVLSFANGVLSSITVTEPKSGVSTMPQATTRSAVYATISNKGLVLNVTTYSTLSAMLSGVATTAEIVKSLKQKPAQQQVVKSSLGKETQEQTQQRIQRALAAKQANAGGVGGVDKQKIYDELNKARNPIKSGKELAKIANTKQKELVKCGAIMMKGNQEWEELVKYLQGGDFTYDAFDAFSDVLKERDYSTCLTKIRALANRLNIVNGVIHLIK*>PLE3_ORF_21MTTTTNINTTFEISAVIAEYKAGFEQYKADNKQYNADAYRRKIESINSDAALTNGAFNQFAYGSQMFEGKTLTEIAEMLKTIPVKDSSREDENGLIFPHVTLQLVSPTTPAQYYGLIAECVKLGFEVCPDWRLHVGTGRQFPACRLVRQSSWYKPHNEKLMESRIAEAEKQEAERLKAEYFNEHRVQAYVQAAQNRFMAQQAQQAAISLSAAISRELYASSGLSDDDLAIVNQSDVWAFNTLAAQLAEKDPNVISAALTGAGFVKGKHKLSDGKNVTLWVKDGADVADVTATDSKFI*>PLE3_ORF_22MKMTAEQFNEHLAHIVKCWAEFGYEIETWEEFLAAELKCIELQAELAAEAEQRVQYALNSLKTLQLK*>PLE3_ORF_23MKYSDTIINKTFTTDKGDKIVKAVTLHAIKQGMLQFKLARVLAPAMGGVVDGMASKDRSLLYATVFNLIAAKATEELFEELQTLLLGSILDSSGEPLETVERINTYFANTSIHQFDLLVWLFEKQLLEPLLKSSALSGFMPKLKTIADNFMQENKEVE*>PLE3_ORF_24MADLFPMPTVSVIGEWLEVMRGDIEDNIEQILTLYLKQFPNTPTQDLLTKTFAYLDFITDTLTKHQRLLTMAESRIEELQTLLAAPDIPESKRVELEKTLYSLKQQKQGGKKQQAVTRWLTKHGVEVSEIENWALYISAESGLTVNDIYNKPWDAFIKLNNTIAVRTMISEAIQHDARHQQQ*>PLE3_ORF_25MMKTLKKRMMLLVTQTLPYLKELREDETGNIVNVDNTPSQLKYDITALEDELYRASIATMQNDEAKKLGFEFSAKVDEVAKLLIAQKDNLDLQEVIFDFLVEFPEYSEAFK*>PLE3_ORF_26MSNAGVVEKFVYENIIDNRKAVRGMRKFEEMTRKHSAKITKLFEDTEKSKQRAIKNTEKEEVKAATKKLTAEQKAAAQAEKLKAQEAAKLKKFNDWKLAQFRSAAFERLSLEQKMELKRVLSAKRSEEEIREEYRQTTAMMRRENQRRAAYERKQSKINNSGVKGGLTAGATGGGIVALAGNPAALAAAAVIGGGAMAINSGSQQFRDTKEGANLVGLDYNEFAQLANGLIAVTETIPDVSTAADKIKDLLDRSGEVMAETVFDSDKGEFDKGEGSILANLLLKQGVITGDKESLNNFMNQSPDKFIESVVKATEGLDAKQQAFVLEAFGSDFYNIIRGIQTNRQGFEAGKQNAVQFNTAEMTAATEFNKSVASMVSAISNADLNIFKSFTEFLSPSSLAMFGKLGELLNSIATLLGYTLSGALNLLSPAINLVLDGLNLLLVGLTNVQRYITELTKYVSDGLTIATEWIKNSLRGMLHDILNVLPDFAKKALGISTVEQTPTTPTTTTAPTTPKPYNMTSPTTYGGYSGYGTQNPTTTTNPTNTTATVNLVVDGNTLATAVVNSAVGQEGVKQIMHRSRPY*>PLE3_ORF_27MQLNKQASKGAFNQIKTLTTLLKRSDKHGFEVGVLQDKKHSSGFSIVGIAWVTHSGSGNIPARAWVYISNAMHKQRRNKAFKAEFKKFLRGKVTLNTVCNVLGNKWVDDDKTVLGDPLILMPNSPQWAARKGKNTPLVETGELRDSIDHKAI*>PLE4_ORF_1MRKQSQSLKPVVVTARPSEIIRFACHSKHLTIKQCVRLRLLAARLEYRQSVI*>PLE4_ORF2MFKVLIHERSKDVKILPDGGFDLTVLLFGVFVPLWRGQYKRALSYLAIVICTLFVAWVFIPFFSNKHYIKDLLEKDGYLLAEDYTGIKPVIPNTSPLMIMLKDFVSWFMFIHVLLGVLGLFILGDLLLAVTIIALFGLGLALRYHRALAKHQAITKYNEVSNAA*>PLE4_ORF_3MLLDQDYIDSNFRKYHVNTIKKWCSGECRGLTEELALNSLDVLLHVEDSTLTEIAESTGLTLETVAKLNAKLVLS*>PLE4_ORF_4_(integrase)MPKLYIFRRVSSKRQTNKTGLKIQVRPETVERLLSEHGLSEVVDLGAEVASASKGKHIEEGSILKRFLMKCERGEIEQGSILAVYSLDRLSRLEKLGLAKSLIYLPITNNGVSIFGEIDNFLYKPHDVLSEILSSLVFERAAEESRAKKNRNIDSLAANLAQYQRDGQYTTKLGKSPSWINHKTGKLNEEAVLWREVINCYTSGQYKNLSALHTAMQLAIPYNTLSKVINNAHKTLIGTKVIKGVELAEFYEPLLTQEEYNRLLIYVRQMGTGRGKAIATTKTENIWLLKGLGECPECGSLCGSFKNGASVKDKVRYNYRCNAAINKHCDSPTYNLNPIEAIVTLLSADYLHEVSQRETNTELNAKIEAMQVEVERLETQLDQATQALNTFFSPELAQAIPKYREQLETAQNELQTLLALASNHTPINESLLSAEIILDKANPERMKLVKLLQGCINRVIVTRKSVKKGDIRFNPKIYTGCDYSLLCVRVEFIDGNIRVFDSVLRDNLRLNLSVSNGTRSIGLENFHRFSRRLREYDLIKYCVINNRKLKIT*>PLE4_ORF_5MKITRLQREFIGEQFHTPKGGTLTVTGITDQTSGRNAVFTVECSICSVDEVLFPDGFTSTKSNLVCNERVPCPCSGRYKYSPNQYHILVQRNCAQKGYTLLEFGAESGEWFGAAKTPITLLNPKTGRTWTTTVYGFLNT*>PLE4_ORF_6MNLLDEFKPVLKIKRQCIGNCAMYRVLLAVGYTKEELQSGYGIHQPLISLIPIGAESEINSFLDGKLEIFNALLQQVGFDAMRYTLQQWFAGLEKDGRYMDYSLMDSSWENVKKSVIPLVLNPEVKFEYV*>PLE4_ORF_7MLKPLPIQSSTQGRVTKRKEINVLCAEVTQEKIAAVDKIKGKIPKMIKQLQDIIDHPDTTDSNRMKAIAQLTAMLERWESDLTKIKNDEEYLDTTTEQQQQQPTTTRKNSSTVDIMALLEKDGK*>PLE4_ORF_8MTDLKSKKLIQIQNEIFSLCKILMKQHYRSNKKTAAIVAMLGLNLTGSQVVEMMQEIEGEKVSLSSVHKARERYRPIVKMLQEETNRLYSLHGFI*>PLE4_ORF_9MQHNTPYLPHQSLQLQSRLIKLYQNVGLFVPKLTFCFSVDNAETDTHYYNVWFSSNVDGELIIGECNTYEKATLIAQQWLDNTTDEDLLHLVQSVLSRGYTDGFSECVEGVRQADKLTSNELLVLECLRPQLDFEEGSESGQYPWSNTDISDKTGIHRSNLHRVLKSLVNKGLIVEETVKRDVWASLKKCGHVQKDVLVYYPVATREEDLEVMENWHEWRAIRQKEAMQKWRAMLSS*>PLE4_ORF_10MKYEIPPSLNLKELPLTTQYQLNRMLNGEIRPSAIRRNKANYKLKGDKDKVFENGLAVRLFNLIREYNNVESVESEEV*>PLE4_ORF_11MKIQLKHPYDKLAGELADLTGLSPTQLTIQLMQELQTKISETSAQEEAKENKNNVKVLPN*>PLE4_ORF_12MSKFYQINTTLLESNEAVNKQTGDVVPLSPETKLVYAYMLNQYRMHRKYGNRRYTESWDKIFTVCCDVAAQKQKKLAKELTTLGLIEVIGNKNAYKVVHSVESIMEAWELTNSKLNTFKDPTQTTKRKESKKQRLNKWEEDKRSKGEWKDFKEPHLEEMQDCNGAESYFTEQPPVEAYEQDYRPIAGDAVNFGVLADIDISDTPTSDQWDCPPEDEPESLTPAKDEPQEVVEVVTPQVVEHVTPVVCGDKSKLLKCFRPNTDISEIDISGKGIGFFAKPLHNKECDKGTARQYLKVIKAYYWDQYNPDDATIDKILGWLVCEVCGNYTDDRGYCSNPNCSTNALPF*>PLE4_ORF_13MSFTKSEILEALQSNLEITNINCNTATLKFKYDNTPFEILLSKMCIKSKNKGFAGSYRTFKSLPELVQLVKSCERFILGDGLVDTIPDLLYKAKVRDAQFIEDCYYKESAYYKLKRELSNNSDYAKKSFKEIKDSCDDFKNWWDRIALPDNWEDFKTWCDTNNCSTQIALELILRRLRFLPSDSGVKAVRFCNIFYWYNGTYRNAKGKTHYQSKNLLHVIRLQDSFNPLEGANIDDYSLLPTDKATLTKEIAMFLSNTEFYKLDMQAYTKGYKDSYVLDTKAL*>PLE4_ORF_14MKYFQIDELTLNAMLRITTIESLTPEQRIELIKAHLLNIKTPSDDNEPWDEF*>PLE4_ORF_15MYQIEDGQAYIIVSRAEFDIYTALNDEGKKAYFDELTHNRPIYSLIED*>PLE4_ORF_16MNIQDVTAAIDLLTIAYVFIKSHPYYIEFTQYLLNQ*>PLE4_ORF_17MTENQTLKGSVDPLPTITLYASGVPVEIAGRIISKSKNSVNYMLYGYRLVDLITHPVINANKHSKQRLYKNLKEIDPIIRNSRQLYTLCPDFLQLWVNRLQQPKLSTDAYKPAENSCYGNILNPEMSRRVKRKRSEEIWPDVLNSFVGVRKITINGNTYYLDKYEHRQYLKMTAGKPAFTRWVNETLFEVWLIAAQPKYRKVKVNHYNEPVKLATYEDFTNSMYEHKLNTNVPQQTEEDY*>PLE4_ORF_18MYQAEFKTKEGARYTVVFTQYVCAMPNPDTLHVTSNRFDFKITRIKNDRSITQDVNFNLKIDGKVIPATQYTVSGQNSKRTSKQTDVILSNYYNWVDLI*>PLE4_ORF_19MPYYSKNAERAALIGCTLPQLKKLNVQFLNIKYRCGKLKGYENTKFKLGTFDDFIEWIASQGGYKTSPHGHTYHVSRIRDQGDYAYNNIRWCSHQENLKETAHFWLVTDIKTSRQELFQGNLTTKLKELFDVSGYGAYKAAESGRLYKGRFKIDKLSI*>PLE4_ORF_20MITIELLNARLALLSPTERKALRLMLTTNLTNAEINKELGVSHNYVYWLKNESPHAETVQMFLNLNNKFKETK*>PLE4_ORF_21MITINLKLGNNESITRTYSQISIEESRIILDTPMESVNQTVLSFANGVLSSITVTEPKQGISTMPQATTRSAVYATISNKGLVLNFTTYATLSVMLSGISANAEIVKGLKQKPAQQQVVKSAALGKETPEQTQQRLQRALAAKQSKQANTGVGGVDKQKIYNELNKARNPIKSGKELAKIANTKQTELVKCGAIMKGNSEWEELVKYLQGGDFTFDAFDAFSDVLKERDYSTCLTKIRALASRLNIVNGVIHLIK*>PLE4_ORF_22MTTTTNIFDLAAVIAEYKAGFEQYKADNKQYNADAYRRKIESINSDAALTNGAYRDFAYGSQLFEGDKTLTEIAEMLKTRTAQTEREDEQGFIYPHVVIQFVSPMTATQYYGLIAECVKLDFEVCPDWRLHVGTGRQFPACRLVRKAEWYKPHNEKLMAERIAEAEKQEAERLKAEYFNEHRVAAYVEQAQRKFMAAQAQQAAISLSAAISRELYATSGLGDDDLAVVAQSDVWAFNTLAPQLQEKDPNVISAALTGAGFVKGKHKLSDGKPATLWVKANADVTATDSKFI*>PLE4_ORF_23MKMTAEQFNERLAHIVKCWAEFGYELETWEEFLAAELKCIEFQAQQAAEAEQRVQYALNSLKTLQLK*>PLE4_ORF_24MKYSDTIINQTFTTDKGDKIVKAVTLHAIKQGMLQFKLARVLAPAMGGVVDGMASADRSLMYATVFNLIAAKATEELFEELQTLLLGSILDSSGEPLETVERINTYFANTSIHQFDLLVWLFEKQLLEPLLKSSALSGFMPKLKTIADNFMQENKEVE*>PLE4_ORF_25MADLFPMPTVSVIGEWLEVMRGDIEDNIEQILTLYLKQFPNTPTQDLLTKTFSYLDFITDTLTKHQRLLTMAESRIEELQTLLAAPDIPEPKRVELEKTLYSLKQQKQGGKKQQAVTRWLTKHGIEVSEIENWALYISAESGLTVNDIYNKPWDAFIKLNNTIAVRTMISEAIQHDARHQQQQ*>PLE4_ORF_26MNFVITNRQNFIKLCVGVVCYWFSIVTLYLYTFTSLQNAFFPITISLLIHLKIYRVLHKKYPCIKEIKTKVIVKKYIEEDNGKLVDLKEWKEQKTNDENIKKADDVTSNTNPTLFKRTKGR*>PLE4_ORF_28MSNAGVVEKFVYENIIDNRKAVRGMRKFEEMTRKHSAKITKLFEDTEKSKQRAIKNTEKEEVKAATKKLTAEQKAAAQAEKLKAQEAAKLKKFNDWKLAQFRSAAFERLSLEQKMELKRVLSAKRSEEEIREEYRQTTAMMRRENQRRAAYERKQSKINNSGVKGGLTAGATGGGIVALAGNPAALAAAAVIGGGAMAINSGSQQFRDTKEGANLVGLDYNEFAQLANGLIAVTETIPDVSTAADKIKDLLDRSGEVMAETVFDSDKGEFDKGEGSILANLLLKQGVITGDKESLNNFMNQSPDKFIESVVKATEGLDAKQQAFVLEAFGSDFYNIVRGIQTNRQGFEAGKQNAVQFNTAEMTAATEFNKSVASMVSAISNADLNIFKSFTEFLSPSSLAMFGKLGELLNSIATLLGYTLSGALNLLSPAINLVLDGLNLLLGGLTNVQRYITELTKYVSDGLTIATEWIKNSLKGMLHDIINALPDFAKKALGISTVEQTPTTPTTTTAPTTPKPYNMTSPTTYGGYSGYGTQNPTTTTNPTNTTATVNLVVDGNTLATAVVNSAVGQEGVKQIMHRSRPY*>PLE4_ORF_27MMKTLKKRMMLLVTQTLPYLKELREDETGNIVNVDNTPSQLKYDITALEDELYRASIATMQNDEAKKLGFEFSAKVDEVAKLLIAQKDNLDLQDVIFDFLVEFPEYSEAFK*>PLE4_ORF_29MQLNKQASKGAFNQIKTLTTLLKRSDKHGFEVGVLQDKKHSSGFSIVGIAWVTHSGSGNIPARAWVYISNAMHKQRRNKAFKAEFKKFLRGKVTLNTVCNVLGNKWVEDDKTVLGDPLILMPNSPQWAARKGKNTPLVETGELRDSIDHKAI*>PLE5_ORF_1_MRKQSLKPVVVTAQPCEIINFVNHSKHLSIKQCVRLRLLAARLEFHQTSI*>PLE5_ORF_2_MFKVLIHERSKDVKILPDGGFDLTVLLFGVFVPLWRGQYKRALSYLAIVICTLFVAWLFIPFFSNKHYIKDLLEKDGYLLAEDYTGIKPVIPDTSPLMIMLKDFVSWFMFIHVLLGVLGLFILGDLLLAVTIIALFGLGLALRYHRALAKHQAITKYNEVSNAA*>PLE5_ORF_3_MLLDQDYIDSNFRKYHVNTIKKWCSGECRGLTEELALNSLDVLLHVEDSTLTEIAESTGLTLETVAKLNAKLVLS*>PLE5_ORF_4_IntegraseMPKLYIFRRVSSKRQTNKTGLKIQVRPETVERLLSEHGLSEVVDLGAEVASASKGKHIEEGSILKRFLMKCERGEIEQGSILAVYSLDRLSRLEKLGLAKSLIYLPITNNGVSIFGEIDNFLYKPHDVLSEILSSLVFERAAEESRAKKNRNIDSLAANLAQYQRDGQYTTKLGKSPSWINHKTGKLNEEAVLWREVINCYTSGQYKNLSALHTAMQLAIPYNTLSKVINNAHKTLIGTKVIKGVELAEFYEPLLTQEEYNRLLIYVRQMGTGKGKAIATTKTENIWLLKGLGECPQCGSLCGSFKNGASVKDKVRYNYRCNAAINKHCDSPTYNLNPIEAIVTLLSADYLHEVSQRETNTELNAKIEAMQVEVERLETQLDQATQALNTFFSPELAQAIPKYREQLETAQNELQTLLALASNHTPINESLLSAEIILDKANPERVKLVKLLQGCINRVIVTRKSVKKGDIRFNPKIYTGCDYSLLCVRVEFIDGNIRVFDSVLRDNLRLNLSVSDGTRSIGLENFHRFSRRLREYDLIKYCVINNRKLKIT*>PLE5_ORF_5_MKITRLQREFIGEQFHTPKGGTLTVTGITDQTSGRNAVFTVECSICSVDEVLFPDGFTSTKSNLVCNERVPCPCSGRYKYSPNQYHILVQRNCVQKGYVLLGFGGEVGEWFGASKTPITLLNPKTGRTWTTTVYGFLNT*>PLE5_ORF_6_MTAQTFNFEYDEYNRLLLPLQYHDITCAFVDALGFRVEDLVGQSNQVRMSLISLIPPQTIVEIKQLVELHHDYFKRWLTEENKCIFNDILHNFIKAYTTTTAKANTYGSGITWQAINSETFKLMIELIDSK*>PLE5_ORF_7_MSNVYPVYKSNDNIKASLDNHLDTLLEAAKGSNKALGRINKAYDGIMLQLIKACKGEIELTKGQSDAIKIVLNEQKHLSKVVADLEELLKKVQAVEAVEGGQQPTTTPNTPPKPFDPRQFKSTKF*>PLE5_ORF_8_MSSKILSKIQNDIIGLGMSLMSETRTNNVTKLVVCLSGLNIPRATIANIVKAETGTTLSVNRITKIRSTYNSIVKTLSEETDRLYQFHEII*>PLE5_ORF_9MQHNTPYLPHQSLQLQSRLIKLYQNVGLFVPKLTFCFSVDNAETDTHYYNVWFSSNVDGELIIGECNTYEKATLIAQQWLDNTTDEDLLHLVQSVLSRGYTDGFSECIEGVRQADKLTSNELLVLECLRPQLDFEEGSESGQYPWSNTDISDKTGIHRSNLHRVLKSLVNKGLIVEETVKRDVWASLKKCGHVQKDVLVYYPVATREEDLEVMENWHEWRAIRQKEAMQKWRAMLSS*>PLE5_ORF_10MKYEIPPSLNLKELPLTTQYQLNRMLNGEIRPSAIRRNKANYKLKGDKDKVFENGLAVRLFNLIKEYNNVESVESEEV*>PLE5_ORF_ORF_11MKIQLKHPYDKLAGELADLTGLSPTQLTIQLMQELQIKISETNAQEEAKENNNNVKVLPN*>PLE5_ORF_ORF_12MSKFYQINTTLLESNEAVNKQTGEVVPLSPETKLVYAYMLNQYRMHRKYGNRRYTESWDKIFTVCCDVATQKQKKLAKELTTLGLIEVIGNKNAYKVVHSVESIMEAWELTNSKLNTFKDPTQTTKRKESKKQRLNKWEEDKRSKGEWKDFKEPHLEEMQDCNGAESYFTEQPPVEAYEQDYRPIAGDTVNFGVLADIDTSGAPTSDQWDCPPEDEPESPTPAKDEPQEVVEVVTPQVVEHVTPVVCGDKSKLLKCFRPNTDISEIDISGKGIGFFAKPLHLKECDKDTARQYLKAIKAYYWDQYNPDNATIANMVSWLTCSVCSNLTNELGYCLDPNCSSNNLPF*>PLE5_ORF_ORF_13MSFTKSEILEALQSNLEITNITCNTATLKFKYENTPFEILPSKMCIKSKNKGFAGSYRTFKSLSELVQLVKSCERFILGDGLVDTIPDLLYKAKVRDAQFIEDCYYKESAYYKLKRELSNNSDYAKKSFKEIKDSCDDFKNWWDRIDLPDNWEDFKTWCDTNNCSTQIALELILRRLRFLPSDSGVKAVRFCNIFYWYNGTYRNAKGKTHYQSKNLLHVIRLQDSFNPLEGANIDDYSLLPTDKATLTKEIAMFLSNTEFYKLDMQAYTKGYKDSYVLDTKAL*>PLE5_ORF_ORF_14MQQFTIDSITLAAMIRICTIETLTEAQRLELIKAHLLNIKTPTTDEWDEF*>PLE5_ORF_ORF_15MYQIEDGQAYIIVSRAEFDIYTALSDAGKKEYFDELTHNRPIYSLIED*>PLE5_ORF_ORF_16MNIQDVTAAIDLLTIAYVFIKSHPYYTEFMQYLLLNQQ*>PLE5_ORF_ORF_17MNPDYVKLKLKQGQIDSINKKIAKLAKKNTPNAVVKKLTTQCLQGLLTYDHEVKVLMEYVVANADCFVDEQVKCAKKYLAAKTAPYPILFLMDFYNAGSSDGWNYKNVLLGI*>PLE5_ORF_ORF_18MYQVEFKTKEGARYTVVFTQYVCAMPNPDTLHVTSDRFDFKITRIKNDRSITQDVNFNLKIDGKVIPATQYVVSGQNSKRTSKQTDVILSNYYNWVDLI*>PLE5_ORF_ORF_19MPYYSKNAERAALIGCTLQQLKKLNVQFLNIKYRCGRLKGYETCQFKLGTFDDFIEWIASQGGYKTSPHGHAYHVSRIRDQGDYEYSNIRFCSHAENLRETAHFWLVTDIKTSRQELFQGNLTTKLKELFDVSGYGAYKAAESGRLYKGRFKIDKLSI*>PLE5_ORF_ORF_20MITIELLSSKLAMLSPTERQALRLMLTTNLTNSEINKELGVSHNYVYWLKNESPHAETVQMFLQLNNKFKETKE*>PLE5_ORF_ORF_21MITINLKLGNSESITRTYSQISIEESRIILDTPTSTVNETVLSFANGVLSSITVTEPKQGVSTMPQATTRSAVYATISNKGLVLNFTTYAILSAMLSGISANAEIVKGLKQKPAQQQVVKSAALGKETPEQTQQRLQRALAAKQSKQANTGVNKQQIYDELNKARNPIKSGKELAKIANTKQTELVKCGAIMKGNSEWEELVKYLQGGDFTYDAFDAFSDVLKERDYSTCLTKIRELANRLNIVNGVIHLIK*>PLE5_ORF_ORF_22MLKTRTAQTEREDEQGFIYPHVVIQFVSPMTATQYYSLIGECIKLGFEVCPDWRLHVGTGRQFPACRLVRKAEWYKPHNEKLMESRIAEAEKHEAERLKAEYFNEQRVQAYVEQAQRKFMAAQAQQAAISLSAAISRELYASSGLSDDDLAIVNQSDVWAFNTLAAQLQEKDPNVISASLQSLGYAKGKHKLSDGKQATLWVKDGADVTALTLESKYIQ*>PLE5_ORF_ORF_23MKMTAEQFNERLAHIVKCWVEFGYEVETWEEFLAAELKCMEFQAELVAEAEQRVQYALNSLKTLQLK*>PLE5_ORF_ORF_24MKYSDTIINKTFTTDKGDKIVKAVTLHAIKQGMLQFKLARVLAPAMGGVVDGMASKDRTLMYSTVFNLIAAKATEELFEELQTLLLGSILDSSGEPLETVERINTYFANTSIHQFDLLVWLFEKQLLEPLLKSSALSGFMPKLKTIADNFMQENKEVE*>PLE5_ORF_ORF_25MTDLFPMPTVSVIGEWLEVMRGDIEDNIEQILTLYLKQFPNTPTQDLLTKTFTYLDFITDTLTKHQRLLTMAESRIEELQTLLAAPDIPEPKRVELEKTLYSLKQQKQGGKKQQAVTRWLTKHGVEVSEIENWALYISAESGLTVNDIYNKPWDAFIKLNNTIAVRTMISEAIQHDARHQQQQQ*>PLE5_ORF_ORF26MNFVITNRQNFIKLCVGVVCYWFSIVTLYLYTFTSLQNAFFPITISLLIHLKIYRVLHKKYPCIKEIKTKVIVKKYIEEDNGKLVDLKEWKEQKANDENIKKADDVTSSTNPTLFKRTKGR*>PLE5_ORF_ORF_27MMKTLKKRMMLLAAQTLPYLKELREDETGNIVNVDNTPSQLKYDITALEDELYRASIATMQNDEAKKLGFEFSAKVDEVAKLLIAQKDNLDLQEVIFDFLVEFPEYSEAFK*>PLE5_ORF_ORF_28MSNAGVVEKFVYENIIDNRKAVRGMRKFEEMTRKYSAKITKLFEDTEKSKQRAIKNTEKEEVKAATKKLTAEQKAAAQAEKLKAQEAAKLKKFNDWKLAQFRSAAFERLSLEQKMELKRVLSAKRSEEEIREEYRQTTAMMRRENQRRAAYERKQSKINNSGVKGGLTAGATGGGIVALAGNPAALAAAAVIGGGAMAINSGSQQFRDTKEGANLVGLDYNEFAQLANGLIAVTETIPDVSTAADKIKDLLDRSGEVMADTVFDSDKGEFDKGEGSILANLLLKQGVITGDKESLNNFMNQSPDKFIDSVVKATEGLDAKQQAYVLEAFGSDFYNIVRGIQTNRQGFEAGKQNAVQFNTAEMTAATEFNKSVASMVSAISNADLNIFKSFTEFLSPSSLAMFGKLGELLNSIATLLGYTLSGALNLLSPAINLVLDGLNLLLGGLTNVQRYITELTKYVSDGLTIATEWIKNSLKGMLHDILNALPDFAKKALGISTVEQTPTTPTTTTAPTTPKPYNMTSPTTYGGYSGYGTQNPTTTTNPTNTTATVNLVVDGNTLATAVVNSAVGQEGVKQIMHRSRPY*>PLE5_ORF_ORF_29MQLNKQASKGAFNQIKTLTTLLKRSDKHGFEVGVLQDKKHSSGFSIVGIAWVTHSGSGNIPARAWVYISNAMHKQRRNKAFKAEFKKFLRGKVTLNTVCNVLGNKWVDDDKNVLGDPLILMPNSPQWAARKGKNTPLVETGELRDSIDHKAI*
